# Enhancing biomedical signals through genetic algorithm optimized Exponentiated transmuted weibull denoising techniques

**DOI:** 10.1038/s41598-026-46088-7

**Published:** 2026-04-25

**Authors:** M. A. Sayedelahl, R. M. Farouk, A. M. Adam

**Affiliations:** 1 Faculty of Computers and Information, Damanhour University, 22511 Damanhour, Egypt; 2 Faculty of Science, Zagazig University, Zagazig, Egypt; 3 Faculty of Science, Mansoura University, Mansoura, Egypt

**Keywords:** Biomedical signal denoising, Exponentiated Transmuted Weibull Distribution, Independent Component Analysis, Genetic Algorithm, EEG, ECG, Medical imaging, Sparsity constraints, Epigenetics analysis, Imaging, Software

## Abstract

Biomedical signals are frequently corrupted by physiological and environmental noise, which obscures diagnostically relevant features and complicates clinical interpretation. This study introduces a denoising framework that integrates the Exponentiated Transmuted Weibull Distribution (ETWD) with Independent Component Analysis (ICA) to model complex, non-Gaussian noise patterns. The ETWD’s tri-parametric structure $\:\left(\boldsymbol{\alpha\:},\boldsymbol{\beta\:},\boldsymbol{\lambda\:}\right)$ generalizes eleven classical distributions, enabling data-driven adaptability beyond conventional heavy-tailed models. A novel score function derived from ETWD is embedded within the FastICA algorithm, with parameters optimized via a Genetic Algorithm (GA). Sparsity constraints in the wavelet domain are applied to preserve transient signal features while suppressing noise. The framework is evaluated on electroencephalogram (EEG), electrocardiogram (ECG), and medical imaging datasets using standardized protocols with 70/30 development/test splits and 10 independent runs. Results demonstrate statistically significant improvements over conventional methods (Gauss, Pow3, Skew, Tanh). For EEG, Sparse ETWD achieved SNR of 7.77 ± 0.07 dB ($\:\boldsymbol{p}<0.01$) and improved epileptic spike detection accuracy from 71% to 94%. For ECG, ETWD achieved SNR of 21.34 ± 0.12 dB ($\:\boldsymbol{p}<0.01$) and improved R-peak detection F1-score from 0.89 to 0.97. For medical images, after correcting the evaluation protocol for normalized data (PSNR = $\:-10{\mathbf{l}\mathbf{o}\mathbf{g}}_{10}\left(\mathrm{MSE}\right)$), ETWD achieved 32.01 dB under Gaussian noise, outperforming baselines across speckle (29.84 dB) and Rician noise (30.92 dB). Cross-dataset validation confirmed robustness within each modality, and comparison with a convolutional autoencoder under identical conditions showed competitive or superior performance without requiring training data. The framework offers a training-free, computationally efficient alternative to deep learning methods, and its application led to improved performance in downstream tasks such as R-peak and spike detection.

## Introduction

 Biomedical signals, including EEGs, ECGs, and medical imaging data, are essential resources for diagnosing neurological issues, cardiovascular diseases, and structural anomalies^1,2^. Nonetheless, these signals are naturally prone to disruption from various noise sources, such as physiological artifacts (e.g., muscle activity, eye movements), environmental interference, and sensor inaccuracies^2,3^. This noise conceals diagnostically essential characteristics and disrupts automated analysis processes, highlighting the need for sophisticated denoising frameworks to tackle non-stationary and multimodal disturbances^4,5^. Traditional methods, such as wavelet transform^3^, adaptive filtering^4,6^, and empirical mode decomposition^2^, show restricted effectiveness in disentangling intricate signal combinations, especially when faced with heavy-tailed or asymmetric noise distributions that are common in clinical settings^7,8,9^.

Blind Source Separation (BSS) has become a fundamental technique for extracting hidden physiological sources from noisy data without needing prior knowledge of the mixing processes^5,10^. Initial methods, such as frequency-domain BSS^10^ and Gaussian mixture models (GMMs)^8^, focused on linear and stationary mixing scenarios. Modern developments encompass nonlinear and underdetermined systems, as demonstrated by their use in gravitational-wave detection^7^ and convolutional signal separation^4^. In biomedical fields, BSS has demonstrated its effectiveness in eliminating EEG artifacts^1^, reducing ECG noise^3,11^, and performing image segmentation^12^. Even with these advancements, conventional BSS techniques frequently depend on limiting assumptions like Gaussian distribution or linear mixing that hinder their flexibility to biomedical data’s high-dimensional, non-stationary characteristics^5,8^. For example, although tensor decompositions^13^ and variants of ICA improve separability in multimodal situations, they face challenges with non-Gaussian noise and computational inefficiencies in parameter optimization^4,8^. ICA, a fundamental component of BSS, facilitates the retrieval of statistically independent sources from combined signals^5^. Conventional ICA models presume linear combinations and non-Gaussian distributions of sources^5^. Nonetheless, contemporary adaptations—like time-frequency masking^4^ and probabilistic models^8^ have expanded their applicability to underdetermined and non-stationary systems. In biomedical fields, ICA has played a crucial role in reducing EEG artifacts^1^, but its effectiveness is still limited in heavy-tailed noise scenarios because of strict statistical assumptions^5,8^.

Recent advancements seek to address these constraints by combining strong statistical models with optimization-focused frameworks. For instance, sparse representations and time-frequency masking^4^ boost separability in underdetermined systems, whereas fractal transformations^12^ enhance feature retention in medical imaging. Nonetheless, these techniques frequently exhibit limited generalizability across various biomedical modalities or create over-smoothing artifacts^2,14^. ETWD, defined by its tri-parametric framework (scale, shape, transmuted), offers an innovative approach for modeling diverse noise patterns, utilizing its adaptability to represent asymmetric and heavy-tailed distributions. Although ETWD has shown potential in both theoretical and applied settings, its combination with BSS for adaptive biomedical denoising is still insufficiently investigated, highlighting a significant gap in existing research.

### Contributions

This work improves biomedical signal denoising through the following contributions:

1. **Adaptive Noise Modelling via a Generalizing Distribution**: While previous ICA-based denoising methods have employed heavy-tailed distributions (e.g., Laplace, Generalized Gaussian) to model source signals, these models possess a fixed parametric form that may not adapt optimally to the varying statistical properties of different biomedical noise types. We introduce the ETWD as a flexible noise model whose key novelty lies in its tri-parametric structure $\:\left(\alpha\:,\beta\:,\lambda\:\right)$. This structure allows it to generalize eleven classical distributions (including exponential, Rayleigh, and Weibull, as shown in Table 1), providing a data-driven adaptability that static heavy-tailed models lack.
2. **Theoretical Unification via a Generalized Score Function**: We provide a theoretical justification for this advancement by deriving a novel score function $\:{\phi\:}_{l}({u}_{l}\mid\:\theta\:)$ from the ETWD for integration with the FastICA algorithm. We mathematically demonstrate that by constraining its parameters, this score function reduces to those of classical source models (e.g., Laplace), thereby proving that ETWD offers a unifying parametric framework for source separation under heavy-tailed conditions rather than an incremental alternative.
3. **Sparse Code Shrinkage Integration**: Wavelet-domain sparsity constraints are introduced to suppress noise-dominated coefficients while preserving diagnostically relevant signal structures.
4. **Genetic Algorithm Optimization**: A GA is used to optimize ETWD parameters, mitigating local minima and achieving efficient global convergence in parameter estimation.
5. **Multimodal Validation**: The framework’s efficacy is validated on EEG, ECG, and medical imaging datasets, demonstrating statistically significant improvements (*p* < 0.01) in SNR, PSNR, and error metrics compared to conventional filters (Gauss, Pow3, Skew, Tanh).

### Organization of the Document

The rest of the document is organized in this manner:

- **Sect. 2 (Literature Review)** analyzes current methods for signal separation, including BSS and ICA, and discusses noise modeling and the application of deep learning for denoising in biomedical contexts.
- **Sect. 3 (Methodology)** explains the proposed approach, which includes the ETWD formulation, how we estimate parameters using genetic algorithms, and the use of sparsity constraints.
- **Sect. 4 (Results and Discussion)** presents experimental results from datasets of EEG, ECG, and medical imaging. It also includes a comparison with traditional and leading-edge methods.
- **Sect. 5 (Conclusion)** concludes the study by summarizing our contributions and discussing the limitations and future directions for research.

## Literature review

The domains of BSS and biomedical signal denoising have witnessed transformative advancements through innovations in nonlinear modeling, adaptive algorithms, and deep learning. This section synthesizes critical developments, emphasizing their contributions and limitations while contextualizing the necessity of the proposed framework.

### Foundational BSS methodologies and nonlinear extensions

Early BSS frameworks focused on linear mixtures, but recent work has expanded into nonlinear and underdetermined systems. Deville et al.^15^ pioneered methods for bilinear, linear-quadratic, and polynomial mixtures, establishing a foundation for nonlinear BSS. Wang et al.^16^ advanced underdetermined BSS for frequency-hopping signals using time-frequency analysis, demonstrating robustness in dynamic environments. Rawat^17^ further bridged BSS with communication systems, proposing computationally efficient MIMO-PA linearization techniques. These nonlinear approaches, however, faced challenges in computational complexity, prompting Liu et al.^18^ to integrate neural networks with maximum likelihood estimation, enhancing adaptability and convergence in real-time applications.

### Statistical models and ICA advancements

Statistical modeling remains pivotal for noise characterization. Altun et al.^19^ extended the gamma distribution for regression tasks, enriching probabilistic frameworks for non-Gaussian signal separation. In ICA, Agcaoglu et al.^20^ introduced a multi-dimensional joint ICA model with a Gaussian copula, enabling robust multimodal data fusion. Adali et al.^21^ further refined ICA through independent vector analysis (IVA), addressing cross-domain dependencies in fMRI and EEG. Despite these strides, convolutive mixtures persisted as a challenge, mitigated by Sarmiento et al.^22^ via permutation-resistant contrasts based on generalized divergences.

### Wavelet transforms and time-frequency analysis

Wavelet-based denoising has revolutionized biomedical signal processing. Kumar et al.^23^ leveraged stationary wavelet transforms (SWT) to suppress ECG noise, while Madan et al.^24^ enhanced SWT with weighted total variation, preserving cardiac features. For respiratory signals, Pouyani et al.^25^ combined discrete wavelet transforms (DWT) with artificial neural networks, achieving 12.4 dB SNR improvement in lung sound denoising. However, mode mixing has limited performance, as Ashraf et al.^26^ addressed through Variational Mode Decomposition (VMD), isolating intrinsic mode functions in surface EMG with 98% accuracy.

### Deep learning and hybrid architectures

Deep learning has redefined BSS through data-driven adaptability. Wang et al.^27^ employed conditional GANs to denoise ECG signals, achieving a 19.8 dB peak SNR, while Rasti-Meymandi and Ghaffari^28^ designed stacked cardiac cycle tensors for deep ECG enhancement. For EMG, Li et al.^29^ optimized Fractional Adaptive Wavelet Transforms (FAWT), reducing noise by 40% in firefighter training assessments. Narayanan and Abhilash^30^ advanced blind compressive sensing, reconstructing signals from 60% fewer measurements via semi-supervised deep networks. Despite their efficacy, deep models often suffer from over-smoothing, as noted by Chen et al.^31^ in EMG applications.

The synergy between deep learning and optimization algorithms has also been explored. For instance, Sa’adah et al.^32^ compared GA and PSO for parameter estimation. Sun et al.^33^ reviewed how metaheuristics, such as GA, PSO, and differential evolution (DE), are used for tuning parameters in biological data analysis.

In signal denoising, Azzouz et al.^34^ improved the quality of ECG signals by combining tunable wavelet transforms with PSO. Mvuh et al.^35^ used adversarial deep learning to enhance ECG signals in high-noise conditions. Lameesa et al.^36^ discussed how metaheuristic techniques are essential in healthcare, such as in diagnosis, medical imaging, and operations management. They found that combining evolutionary optimization with deep learning can create better solutions for reducing noise in biomedical signals. This combination is a key area for future research.

### Domain-specific biomedical applications

Tailored denoising solutions have emerged for clinical challenges. Vargas and Veiga^37^ introduced noise variation estimates for ECG, reducing baseline wander by 89%. Ranjan et al.^38^ suppressed EEG motion artifacts using adaptive signal denoising, improving cross-correlation to 0.96. In imaging, Chaudhary and Pachori^39^ enhanced MRI clarity via Fourier-Bessel empirical wavelet transforms, achieving 22.5 dB PSNR. Daoui et al.^40^ further secured biomedical data using fractional Charlier-Krawtchouk transformations, enabling zero-watermarking with 99.3% robustness.

### Critical gaps and research imperatives

While existing methods excel in specific contexts, three limitations persist:

1. **Noise Adaptability**: Conventional ICA and wavelet techniques struggle with heterogeneous noise profiles standard in multimodal biomedical data^20,23^.
2. **Computational Efficiency**: Deep learning models, though powerful, incur high computational costs^27,28^.
3. **Generalizability**: Few frameworks unify denoising across EEG, ECG, and imaging modalities^21,39^.

This review underscores the need for an adaptive, computationally efficient model capable of addressing multimodal noise and bridging a gap in our ETWD-GA framework. Our approach advances beyond the state-of-the-art by integrating the tri-parametric ETWD for noise modeling, genetic algorithms for parameter optimization, and sparse constraints for feature preservation, as validated in subsequent sections.

## Context and methodology

Biomedical signals are often contaminated by physiological and environmental noise, which can overwhelm the underlying signal. Effective denoising requires separating these components without distorting diagnostically relevant features. To overcome this problem, we have developed a new framework that merges two powerful tools:

1. **A Smart Noise Model (ETWD)**: A tri-parametric statistical model (ETWD) is employed to characterize the distribution of noise, offering adaptability to various heavy-tailed and asymmetric profiles.
2. **Signal Separation (ICA)**: The FastICA algorithm, utilizing a novel score function derived from the ETWD, is used to separate the observed signals into statistically independent source and noise components.

Traditional fine-tuning systems act like solving a blindfolded puzzle, so we added a genetic algorithm as a kind of computational evolution to automatically find the best settings for our noise model, speeding up the process and boosting accuracy.

We tested our framework using real-world data, measuring how well it cleans up EEGs, ECGs, and other biomedical images.

### Algorithms at the heart of our study

The proposed denoising framework integrates five key components:

#### BSS algorithm

BSS aims to recover latent source signals from observed mixtures without knowledge of the mixing process, making it suitable for real-world biomedical data.

Assume we have a set of independent source signals, represented by the vector.

1 $$\:S\left(t\right)\:=\:{\left[{s}_{1}\right(t),{s}_{2}\:(t),\:.\:.\:.\:,\:{s}_{N}(t\left)\right]}^{T}(t\:=\:1,\:2,\:.\:.\:.\:,\:l)$$

These signals are mixed linearly to create observed signals, denoted by

2 $$\:X\left(t\right)\:=\:{\left[{x}_{1}\right(t),{x}_{2}\:(t),\:.\:.\:.\:,\:{x}_{K}(t\left)\right]}^{T}(N\:=\:K)$$

Mathematically, this relationship can be expressed as:

3 $$\:X\left(t\right)=AS\left(t\right)$$

where $\:\mathrm{A}\:\mathrm{i}\mathrm{s}\:\mathrm{a}\:\mathrm{N}\:\times\:\:\mathrm{N}$ mixing matrix.

The goal of BSS is to recover the original source signals S(t) from observed mixtures $\:\mathrm{x}\left(\mathrm{t}\right)\:$ by finding a separation matrix **W** such that:

4 $$\:\mathrm{U}\left(\mathrm{t}\right)=\mathrm{W}\mathrm{X}\left(\mathrm{t}\right)$$

where $\:\mathrm{W}\:\mathrm{i}\mathrm{s}\:\mathrm{a}\:\mathrm{N}\:\times\:\:\mathrm{N}$ separation matrix and $\:\mathrm{U}\left(\mathrm{t}\right)=\:{\left[{\mathrm{u}}_{1}\right(\mathrm{t}),{\mathrm{u}}_{2}\:(\mathrm{t}),\:.\:.\:.\:,\:{\mathrm{u}}_{\mathrm{N}}(\mathrm{t}\left)\right]}^{\mathrm{T}}\:$ is the estimate of $\:\mathrm{N}$ sources.

Typically, we assume the source signals have zero mean and unit variance, often following a Gaussian distribution. To estimate the unmixing matrix **W**, we commonly employ Independent Component Analysis (ICA). ICA aims to maximize the likelihood of the observed data by adjusting **W**. This involves optimizing the following negative log-likelihood function:

5 $$\:L\left(u,W\right)=\sum\:_{l=1}^{N}E\left[{log}{p}_{ul}\left({u}_{l}\right)\right]-log\left|{det}\left(W\right)\right|$$

where $\:\mathrm{E}[.]$ represents the expectation operator and $\:{\mathrm{p}}_{\mathrm{u}1}\left({\mathrm{u}}_{1}\right)$ is the model for the marginal pdf of $\:{\mathrm{u}}_{\mathrm{l}}$, for all $\:\mathrm{l}=\mathrm{1,2},\dots\:,\mathrm{N}$. In effect, when rightly supposing upon the distribution of the sources, the maximum likelihood (ML) principle leads to estimating functions, which are the score functions of the sources^16^.

6 $$\:{\phi\:}_{l}\left({u}_{l}\right)=-\frac{d}{d{u}_{l}}{log}{p}_{ul}\left({u}_{l}\right)$$

Various ICA algorithms can be used to optimize this criterion, such as FastICA.

The FastICA relies on a contrast function to measure the non-Gaussianity of the estimated sources based on:

7 $$\:{W}_{k+1}={W}_{k}+D(E\left[\phi\:\left(u\right){u}^{T}\right]-diag({E[\phi\:}_{l}\left({u}_{l}\right){u}_{l}\left]\right)){W}_{k}$$

8 $$\:D=diag\left(\frac{1}{{E[\phi\:}_{l}\left({u}_{l}\right){u}_{l}]-E[{\phi\:}_{l}^{{\prime\:}}\left({u}_{l}\right)]}\right)$$

where $\:\phi\:\left(t\right)={[{\phi\:}_{1}\left({u}_{1}\right),{\phi\:}_{2}\left({u}_{2}\right),\dots\:,{\phi\:}_{n}({u}_{n}\left)\right]}^{T}$, valid for all $\:\mathrm{l}\:=1,\:2,\:\dots\:,\:\mathrm{n}$.

#### ICA algorithm

ICA is a core BSS technique that identifies statistically independent, non-Gaussian components from mixed signals. Then, it separates a multivariate signal into its underlying components. ICA aims to find unrelated hidden factors within data^41^.

**Mathematically**, ICA can be expressed as:

9 $$\:{x}_{j}\:=\:{a}_{j1}{s}_{1}+{a}_{j2}{s}_{2}+...+{a}_{jn}{s}_{n},\:for\:all\:j$$

Where j ranges from 1 to n, the time indicator t has been omitted.

To simplify computations, we can assume a zero mean for both the admixture variables and the independent factors. If not, the observed variables $\:{\mathrm{x}}_{\mathrm{j}}$​ can be centered by subtracting the sample mean, ensuring the model is zero-mean. Utilizing a vector-matrix notation, let x represent the arbitrary vector with elements $\:{\mathrm{x}}_{1},\dots\:.,{\mathrm{x}}_{\mathrm{n}}$. s the arbitrary vector with elements $\:{\mathrm{s}}_{1},\dots\:.,{\mathrm{s}}_{\mathrm{n}}$, and A the matrix with elements a_ij_​. The mixing model is expressed as:

10 $$\:\mathrm{x}\:=\:\mathrm{A}\mathrm{s}$$

Alternatively, it can be written as:

11 $$\:\mathrm{x}\:=\sum\:_{\mathrm{i}=1}^{\mathrm{n}}{a}_{i}\:{\mathrm{s}}_{i}$$

The statistical model in Eq. 11 is referred to as the ICA model, serving as a generative model that explains how observed data are generated through the mixing of factors $\:{\mathrm{s}}_{i}$.

While ICA is a powerful tool, its implementation requires sophisticated algorithms and optimization strategies to address challenges like non-convexity. Its versatility has led to applications across various fields, including signal processing, image processing, medical signal analysis, and financial data analysis, where it tackles tasks such as blind source separation, noise reduction, feature extraction, image denoising, and more.

##### The fastICA algorithm

Several methods exist to measure the non-Gaussian data, which are essential for ICA. The FastICA algorithm is a popular and efficient technique for maximizing these measures and extracting the underlying independent components. FastICA is employed in this work due to its computational efficiency.

##### Sparse code shrinkage

Sparse Code Shrinkage is a statistical approach to image denoising that leverages ICA. The method models a noisy image as a linear combination of underlying, non-Gaussian source signals corrupted by additive Gaussian noise.

Given a noisy image, represented as a vector $\:\boldsymbol{z}$ (Eq. 12), the goal is to recover the original, noise-free image $\:\boldsymbol{x}$. To achieve this, the image is decomposed into a linear combination of independent components $\:s$ using an orthogonal transformation matrix $\:\boldsymbol{w}$. The noise component $\:\boldsymbol{n}$ is assumed to be Gaussian and uncorrelated.

12 $$\:\boldsymbol{z}\:=\:\boldsymbol{x}+\boldsymbol{n}$$

The core idea is to apply a shrinkage function to the transformed image $\:Wz$ (Eq. 11) to suppress noise while preserving essential image features. This shrinkage process is based on maximum likelihood estimation under the assumption of Laplacian distributed source components.

This leads to the Sparse Code Shrinkage method^42^, where the Maximum Likelihood solution for the signal involves applying a shrinkage function to the transformed coefficients, assuming a sparse prior (e.g., Laplacian).

The ML solution involves a decomposition that is an orthogonalized version of ICA, expressed as:

13 $$\:Wz\:=Wx+Wn\:=\:s+Wn$$

where $\:\boldsymbol{W}$ is an orthogonal matrix which is the best orthogonal approximation of the inverse of the ICA mixing matrix. The noise term $\:\boldsymbol{W}\boldsymbol{n}$ is still Gaussian and white. With a quietly suitable choice of orthogonal transform, however, the density of $\:\boldsymbol{W}\boldsymbol{x}\:=\:\boldsymbol{s}$ becomes largely non-Gaussian, e.g., super-Gaussian with a highly positive kurtosis. This relies obviously on the original **x** signals, assuming there exists a model $\:\boldsymbol{x}\:=\:{\boldsymbol{W}}^{T}\:\boldsymbol{s}$ for the signal, where the “source signals” or elements of **s** have a positive kurtotic density, in such a case, the ICA transform gives highly super-Gaussian components.

As demonstrated in^42^, assuming a Laplacian density for $\:{s}_{i}$, the ML solution for $\:{s}_{i}$ is given by a “shrinkage function” $\:{\widehat{s}}_{i}\:=\:g\left({\left[\boldsymbol{W}\boldsymbol{z}\right]}_{i}\right)$, or in vector form, $\:\widehat{\boldsymbol{s}}\:=\:g\left(\boldsymbol{W}\boldsymbol{z}\right)$. Function *g*(.) exhibits a characteristic shape, being zero close to the origin and then linear after a cutting value determined by the parameters of the Laplacian density and the Gaussian noise density. Different optimal shrinkage functions can be derived by considering other forms for the densities.

In the Sparse Code Shrinkage model, the shrinkage process is executed in the rotated space, and the estimation of the signal in the original space is obtained by rotating back:

14 $$\:\widehat{x}\:={W}^{T}\:\widehat{s}\:={W}^{T}\:g\left(Wz\right)$$

The Sparse Code Shrinkage method effectively removes noise from images by maximizing the likelihood of the estimated image given the observed noisy data. This is achieved by transforming the image into a domain where its components are sparse, using a rotation matrix learned through a modified FastICA algorithm. The resulting denoised image is obtained by applying an inverse transformation to the processed components. This approach surpasses traditional methods by preserving image details while significantly reducing noise.

#### ETWD algorithm

ETWD is a flexible parametric model for characterizing noise distributions. Its probability density function (PDF) is defined using three parameters (scale, shape, and transmuted). This flexibility allows ETWD to model various data patterns and skewed noise better than classic tools, as demonstrated by its ability to generate a wide range of distributions by adjusting parameter values^43^. The probability density function (PDF) of ETWD, also known as the Exponentiated Transmuted Weibull Probability Density (MWPD), is explicitly defined as:

15 $$\:f\left(x\right)=\frac{\nu\:\beta\:}{\alpha\:}{\left(\frac{{x}_{i}}{\alpha\:}\right)}^{\beta\:-1}{\:e}^{{-\left(\frac{{x}_{i}}{\alpha\:}\right)}^{\beta\:}}\left[1-\lambda\:+2\lambda\:{e}^{{-\left(\frac{{x}_{i}}{\alpha\:}\right)}^{\beta\:}}\right]\times\:{\left[1+\left(\lambda\:-1\right){e}^{{-\left(\frac{{x}_{i}}{\alpha\:}\right)}^{\beta\:}}-\lambda\:{e}^{{-2\left(\frac{{x}_{i}}{\alpha\:}\right)}^{\beta\:}}\right]}^{\nu\:-1}$$

The ETWD is a versatile statistical model defined by three adjustable parameters: Scale (α), which governs the spread of the distribution; shape (β), which controls the skewness and tail behavior (β > 0); and Transmuted (λ), which modifies symmetry (limited to |λ| ≤ 1).

**Fig. 1:**
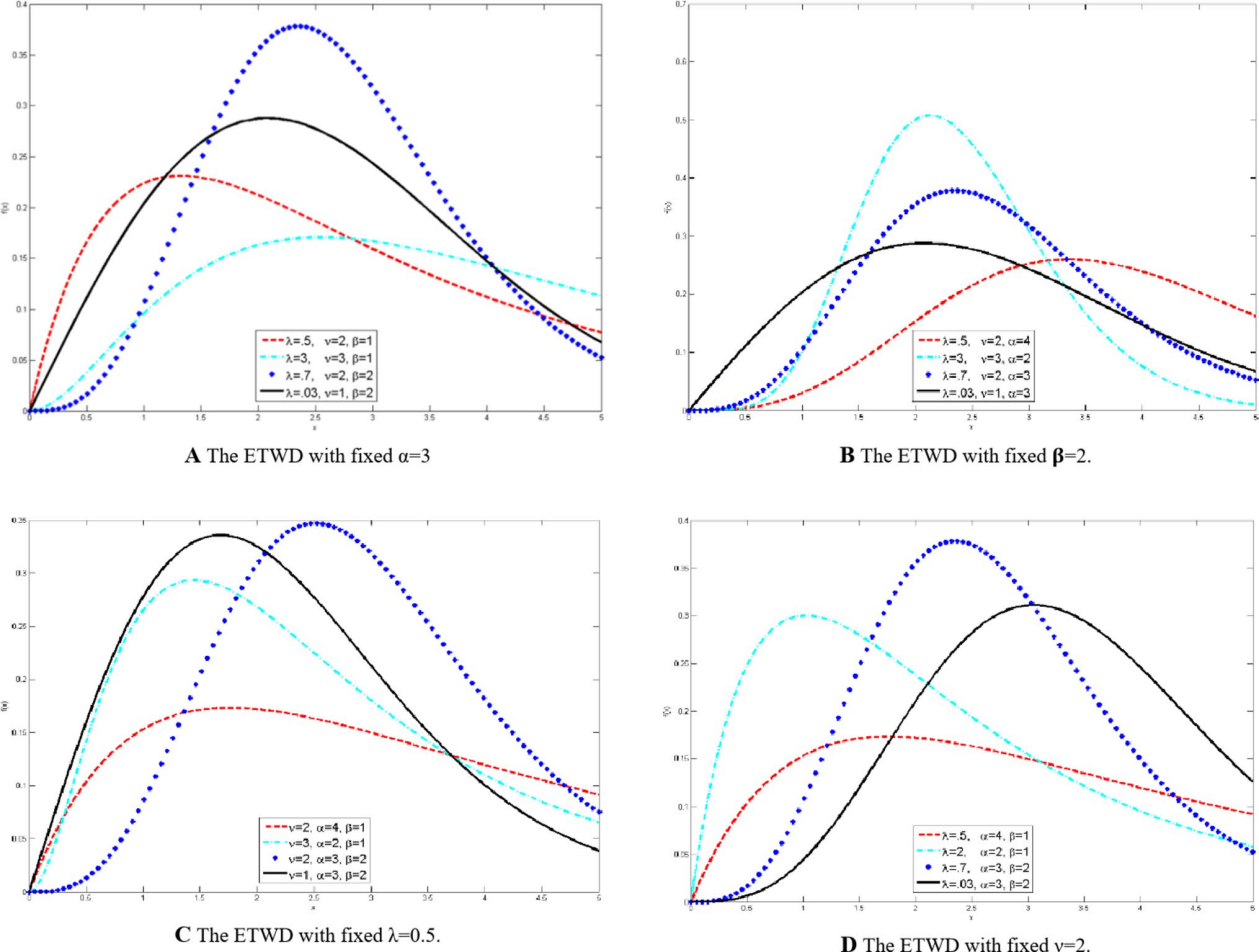
The **ETWD** with different parameters.

The ETWD can be tailored to match diverse data behaviors, from symmetric to heavily skewed patterns, by tuning these parameters. It generalizes eleven classical distributions, including exponential, Rayleigh, and Weibull, as exceptional cases, summarized in Table 1. Figure 1 demonstrates this flexibility visually. Subplots (a-d) display distinct probability density functions (PDFs) generated by varying α, β, and λ. For example, Fig. 1A (fixed α = 3): Increasing β sharpens the peak, while λ adjusts asymmetry; Fig. 1B: By varying β and λ simultaneously, the model transitions smoothly between heavy-tailed and near-symmetric profiles. For instance, higher β values with negative λ skew the distribution left, mimicking real-world scenarios like sensor drift in biomedical; Fig. 1C (fixed λ = 0.5): Altering α and β shifts the distribution’s center and spread; and Fig. 1D: A showcase of extreme parameter combinations, like high β with |λ|=1 producing sharp, highly skewed peaks. Such shapes are critical for modeling abrupt signal anomalies, such as motion artifacts in ECG data.

Corresponding cumulative distribution:

16 $$\:F\left(x\right)={\left\{1+\left(\lambda\:-1\right){e}^{-{\left(\frac{x}{\alpha\:}\right)}^{\beta\:}}-\lambda\:{e}^{-{2\left(\frac{x}{\alpha\:}\right)}^{\beta\:}}\right\}}^{\nu\:},x\ge\:0$$

**Table 1:**
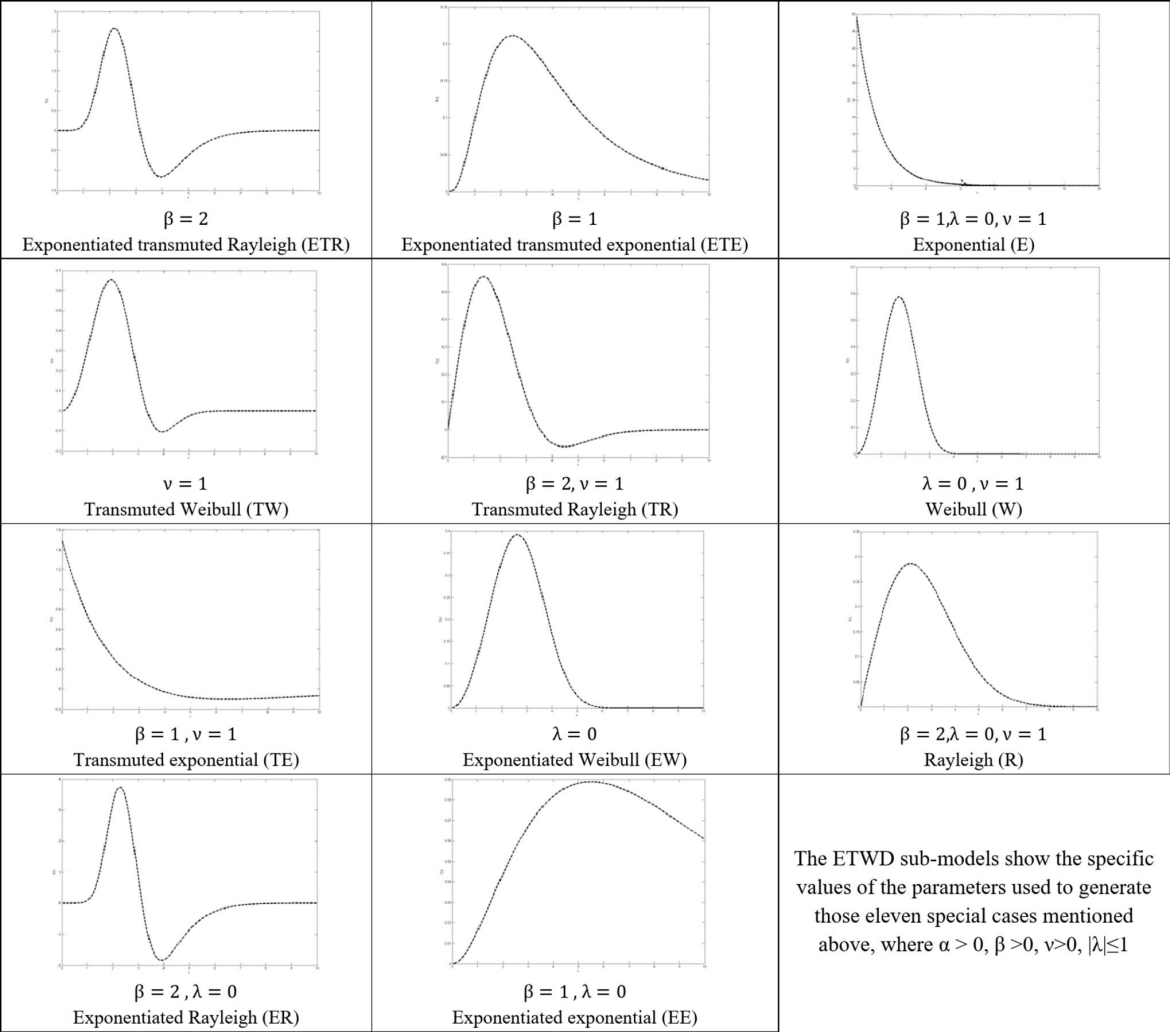
The etwd sub-models.

##### Maximum likelihood estimation (MLE)

MLE is used to estimate the ETWD parameters by maximizing the likelihood function of the observed data.

##### Parameter estimation

The estimation of ETWD parameters employs the Maximum Likelihood approach. Let $\:{\mathrm{X}}_{1},{\mathrm{X}}_{2}\dots\:,\:{\mathrm{X}}_{\mathrm{n}}$ be a sample of size N from an ETWD. Then the log-likelihood function ($\:\mathcal{L}$) is given by:

17 $$\:\mathcal{l}=\prod\:_{i=1}^{n}{f}_{i}\left(x\right)=\prod\:_{i=1}^{n}\left[\frac{\nu\:\beta\:}{\alpha\:}{\left(\frac{{x}_{i}}{\alpha\:}\right)}^{\beta\:-1}{\:e}^{{-\left(\frac{{x}_{i}}{\alpha\:}\right)}^{\beta\:}}\left[1-\lambda\:+2\lambda\:{e}^{{-\left(\frac{{x}_{i}}{\alpha\:}\right)}^{\beta\:}}\right]\times\:{\left[1+\left(\lambda\:-1\right){e}^{{-\left(\frac{{x}_{i}}{\alpha\:}\right)}^{\beta\:}}-\lambda\:{e}^{{-2\left(\frac{{x}_{i}}{\alpha\:}\right)}^{\beta\:}}\right]}^{\nu\:-1}\right]$$

Hence, the log-likelihood function $\:\mathcal{L}=\mathrm{log}\mathcal{l}$ becomes

18 $$\:\mathcal{L}={log}\mathcal{l}={log}\left(\prod\:_{i=1}^{n}\left[\frac{\nu\:\beta\:}{\alpha\:}{\left(\frac{{x}_{i}}{\alpha\:}\right)}^{\beta\:-1}{\:e}^{{-\left(\frac{{x}_{i}}{\alpha\:}\right)}^{\beta\:}}\left[1-\lambda\:+2\lambda\:{e}^{{-\left(\frac{{x}_{i}}{\alpha\:}\right)}^{\beta\:}}\right]\times\:{\left[1+\left(\lambda\:-1\right){e}^{{-\left(\frac{{x}_{i}}{\alpha\:}\right)}^{\beta\:}}-\lambda\:{e}^{{-2\left(\frac{{x}_{i}}{\alpha\:}\right)}^{\beta\:}}\right]}^{\nu\:-1}\right]\right)$$

19 $$\begin{aligned} \:\mathcal{L}=\mathcal{L}={log}\nu\:+{log}\beta\:-\:\beta\:{log}\alpha\:+\:\left(\beta\:-1\right)\sum\:_{i=1}^{n}\mathrm{log}({x}_{i})-\:\sum\:_{i=1}^{n}{\left(\frac{{x}_{i}}{\alpha\:}\right)}^{\beta\:}+\:\sum\:_{i=1}^{n}{log}\big[1-\lambda\:+2\lambda\:{e}^{{-\left(\frac{{x}_{i}}{\alpha\:}\right)}^{\beta\:}}\big]\\+\:\left(\nu\:-1\right)\:\sum\:_{i=1}^{n}{log}\big[1+\left(\lambda\:-1\right){e}^{{-\left(\frac{{x}_{i}}{\alpha\:}\right)}^{\beta\:}}-\lambda\:{e}^{{-2\left(\frac{{x}_{i}}{\alpha\:}\right)}^{\beta\:}}\big] \end{aligned}$$

Hence, the maximum likelihood estimation of α, β, and λ is obtained through the derivatives of L, ensuring satisfaction of the following equations:

$$\:\frac{\partial\:\mathcal{L}}{\partial\:\alpha\:}=0,\:\:\:\frac{\partial\:\mathcal{L}}{\partial\:\lambda\:}=0,\:\:\:\frac{\partial\:\mathcal{L}}{\partial\:\beta\:}=0,\:\:\frac{\partial\:\mathcal{L}}{\partial\:\nu\:}=0$$

20 $$\begin{aligned} \:\frac{\partial\:\mathcal{L}}{\partial\:\alpha\:}=\:-\:\frac{n\beta\:}{\alpha\:}\:+\:\beta\:\sum\:_{i=1}^{n}{\left(\frac{{x}_{i}}{\alpha\:}\right)}^{\beta\:-1}\:+\sum\:_{i=1}^{n}\frac{2\lambda\:{e}^{{-\left(\frac{{x}_{i}}{\alpha\:}\right)}^{\beta\:}}\beta\:{\left(\frac{{x}_{i}}{\alpha\:}\right)}^{\beta\:-1}\left(\frac{{x}_{i}}{{\alpha\:}^{2}}\right)}{2\lambda\:{e}^{{-\left(\frac{{x}_{i}}{\alpha\:}\right)}^{\beta\:}}-\lambda\:+1}+\:\left(\nu\:-1\right)\\ \times\:\sum\:_{i=1}^{n}\frac{\left(\lambda\:-1\right){e}^{{-\left(\frac{{x}_{i}}{\alpha\:}\right)}^{\beta\:}}\beta\:{\left(\frac{{x}_{i}}{\alpha\:}\right)}^{\beta\:-1}\left(\frac{{x}_{i}}{{\alpha\:}^{2}}\right)-2\lambda\:{e}^{{-2\left(\frac{{x}_{i}}{\alpha\:}\right)}^{\beta\:}}\beta\:{\left(\frac{{x}_{i}}{\alpha\:}\right)}^{\beta\:-1}\left(\frac{{x}_{i}}{{\alpha\:}^{2}}\right)}{\left(\lambda\:-1\right){e}^{{-\left(\frac{{x}_{i}}{\alpha\:}\right)}^{\beta\:}}-\lambda\:{e}^{{-2\left(\frac{{x}_{i}}{\alpha\:}\right)}^{\beta\:}}+1}\:\end{aligned}$$

21 $$\begin{aligned} \:\frac{\partial\:\mathcal{L}}{\partial\:\beta\:}\:=\frac{n}{\beta\:}+\sum\:_{i=1}^{n}{log}\left({x}_{i}\right)-n{log}\alpha\:-\sum\:_{i=1}^{n}{\left(\frac{{x}_{i}}{\alpha\:}\right)}^{\beta\:}{log}\left(\frac{{x}_{i}}{\alpha\:}\right)+\sum\:_{i=1}^{n}\frac{-2\lambda\:{e}^{{-\left(\frac{x}{\alpha\:}\right)}^{\beta\:}}{\left(\frac{x}{\alpha\:}\right)}^{\beta\:}{log}\left(\frac{{x}_{i}}{\alpha\:}\right)}{2\lambda\:{e}^{{-\left(\frac{{x}_{i}}{\alpha\:}\right)}^{\beta\:}}-\lambda\:+1}+\left(\nu\:-1\right)\\ \times\:\sum\:_{i=1}^{n}\frac{-\left(\lambda\:-1\right){e}^{{-\left(\frac{{x}_{i}}{\alpha\:}\right)}^{\beta\:}}{\left(\frac{{x}_{i}}{\alpha\:}\right)}^{\beta\:}{log}\left(\frac{{x}_{i}}{\alpha\:}\right)+2\lambda\:{e}^{{-2\left(\frac{{x}_{i}}{\alpha\:}\right)}^{\beta\:}}{\left(\frac{{x}_{i}}{\alpha\:}\right)}^{\beta\:}{log}\left(\frac{{x}_{i}}{\alpha\:}\right)}{\left(\lambda\:-1\right){e}^{{-\left(\frac{{x}_{i}}{\alpha\:}\right)}^{\beta\:}}-\lambda\:{e}^{{-2\left(\frac{{x}_{i}}{\alpha\:}\right)}^{\beta\:}}+1}\end{aligned}$$

22 $$\:\frac{\partial\:\mathcal{L}}{\partial\:\lambda\:}=\:\sum\:_{i=1}^{n}\frac{2{e}^{{-\left(\frac{{x}_{i}}{\alpha\:}\right)}^{\beta\:}}-1}{2\lambda\:{e}^{{-\left(\frac{{x}_{i}}{\alpha\:}\right)}^{\beta\:}}-\lambda\:+1}+\left(\nu\:-1\right)\times\:\sum\:_{i=1}^{n}\frac{{e}^{{-\left(\frac{{x}_{i}}{\alpha\:}\right)}^{\beta\:}}-{e}^{{-2\left(\frac{{x}_{i}}{\alpha\:}\right)}^{\beta\:}}}{\left(\lambda\:-1\right){e}^{{-\left(\frac{{x}_{i}}{\alpha\:}\right)}^{\beta\:}}-\lambda\:{e}^{{-2\left(\frac{{x}_{i}}{\alpha\:}\right)}^{\beta\:}}+1}$$

23 $$\:\frac{\partial\:\mathcal{L}}{\partial\:\nu\:}=\:\sum\:_{i=1}^{n}{log}\left[\left(\lambda\:-1\right){e}^{{-\left(\frac{{x}_{i}}{\alpha\:}\right)}^{\beta\:}}-\lambda\:{e}^{{-2\left(\frac{{x}_{i}}{\alpha\:}\right)}^{\beta\:}}+1\right]+\frac{n}{\nu\:}.\:\:$$

Obtaining estimates for the ETWD parameters requires solving a system of nonlinear equations derived from the log-likelihood function. Due to the complexity of this system, analytical solutions are often intractable. Consequently, numerical optimization techniques are employed. GA provide a robust and efficient approach to this problem by directly optimizing the log-likelihood function without requiring derivative calculations^44^.

#### Genetic algorithm optimization

GA^45^ is employed for robust parameter estimation of the ETWD model. Inspired by natural selection, GA evolves a population of candidate solutions toward optimal settings, effectively mitigating the risk of local optima that gradient-based methods may encounter. The GA configuration, detailed in Table 2, was selected based on preliminary experiments to balance exploration and exploitation while ensuring stable convergence.

**Table 2:** Genetic algorithm configuration parameters.

Parameter	Value
Population Size	100
Selection Method	Tournament (size 3)
Crossover Type	Uniform
Crossover Rate	0.8
Mutation Type	Gaussian
Mutation Rate	0.05
Elitism Count	2
Number of Generations	200
Fitness Function	Negative Log-Likelihood ($\:-\mathcal{L}$)

To evaluate the effectiveness of GA in estimating ETWD parameters, experiments were conducted on simulated datasets featuring diverse parameter combinations. The GA was configured with the parameters listed in Table 2 and run on the development set (70% of the data) to minimize the negative log-likelihood. A validation subset (20% of the development set) was used to monitor convergence and prevent overfitting. The final optimized parameters were then evaluated on the held-out test set (30% of the data). Table 3 presents the actual parameter values, estimated values, and corresponding errors, demonstrating the GA’s robustness and precision.

**Table 3:** Parameter estimation results using ga.

$\:\boldsymbol{\uplambda\:}$	$\:\boldsymbol{\upnu\:}$	$\:\boldsymbol{\upalpha\:}$	$\:\boldsymbol{\upbeta\:}$	$\:\widehat{\boldsymbol{\uplambda\:}}$	$\:\widehat{\boldsymbol{\upnu\:}}$	$\:\widehat{\boldsymbol{\upalpha\:}}$	$\:\widehat{\boldsymbol{\upbeta\:}}$	Err
0.5	2	3	4	0.59	1.86	2.97	4.11	0.02
1	2.5	5.2	6.8	1.16	2.42	5.27	6.80	0.06
3	5.7	1.9	8.2	2.98	5.63	1.98	8.12	0.006

The GA-based approach achieved consistently low error rates across all parameter combinations, highlighting its precision and reliability. To assess the stability and convergence of the GA-based parameter estimation, we performed 10 independent runs and analyzed the evolution of the fitness function (negative log-likelihood) over generations. The results, including convergence trajectories and variability across runs, are presented in Sect. 4.1.

#### Theoretical generalization of the score function

To substantiate the claim that the ETWD provides a unifying parametric framework for source separation, we demonstrate how its derived score function generalizes classical heavy-tailed models commonly used in ICA. The score function for a source estimate $\:u$ is defined as the negative derivative of the log-density:

$$\:\phi\:\left(u\right)=-\frac{d}{du}\mathrm{l}\mathrm{o}\mathrm{g}p\left(u\right)$$

Using the ETWD density from Eq. (15), the resulting parametric score function is:

24 $$\:\phi\:(u\mid\:\alpha\:,\beta\:,\lambda\:,\nu\:)=-\frac{d}{du}\mathrm{l}\mathrm{o}\mathrm{g}{f}_{\mathrm{ETWD}}(u\mid\:\alpha\:,\beta\:,\lambda\:,\nu\:)$$

While the full analytical expression is complex, its behaviour under specific parameter constraints reveals its generalizing nature:

- **Reduction to Laplace Distribution**: The Laplace (double-exponential) distribution, a standard heavy-tailed model in ICA, has a score function proportional to $\:\mathrm{s}\mathrm{i}\mathrm{g}\mathrm{n}\left(u\right)$. By setting the ETWD shape parameter $\:\beta\:=1$ and applying a linear transformation, the ETWD density simplifies to a form whose score function exhibits a piecewise linear behavior around the origin, approximating the Laplacian score. Specifically, as the transmuting parameter $\:\lambda\:$ and exponentiation parameter $\:\nu\:$ are tuned, the function $\:\phi\:\left(u\right)$ transitions smoothly from a near-linear function (Gaussian-like) to a function with a sharp cusp at zero (Laplace-like). This demonstrates that the Laplace score function is a special case of the ETWD-based score function under specific parameter constraints.
- **Generalization of Generalized Gaussian**: The Generalized Gaussian Distribution (GGD) is a flexible model that encompasses both Gaussian ($\:{\beta\:}_{\mathrm{GG}}=2$) and Laplace ($\:{\beta\:}_{\mathrm{GG}}=1$) distributions. Its score function is $\:{\phi\:}_{\mathrm{GG}}\left(u\right)\propto\:\mid\:u{\mid\:}^{{\beta\:}_{\mathrm{GG}}-2}u$. The ETWD score function, with its additional parameters $\:\lambda\:$ and $\:\nu\:$, can model asymmetries and more complex tail behaviors that the symmetric GGD cannot capture. For instance, when $\:\lambda\:=0$, the ETWD reduces to the Exponentiated Weibull distribution, which, depending on $\:\beta\:$, can model a wider range of tail weights than the GGD while also allowing for asymmetry when $\:\lambda\:\ne\:0$.
- **Unifying Framework**: The tri-parametric nature of ETWD decouples the modelling of the distribution’s peak (kurtosis) and its tail asymmetry through independent parameters. This decoupling allows the ETWD score function to morph continuously between the score functions of its eleven special cases (Table 1), including exponential, Rayleigh, and Weibull. Hence, by optimizing $\:\alpha\:,\beta\:,\lambda\:,\nu\:$ via GA, the FastICA algorithm effectively selects the most appropriate source prior from a rich family, rather than being restricted to a single fixed form.

This theoretical property confirms that the ETWD-based score function provides a **richer parametric family** than the standard functions (e.g., tanh, pow3, gauss). It enables the ICA algorithm to adapt its source prior more accurately to the observed data, leading to the performance improvements documented in Sect. 4 Results and discussion.

#### Sparsity constraints in sparse ETWD

Sparsity constraints are applied through a three-stage process to suppress noise while preserving critical signal features:

1. Wavelet Transformation:
  The noisy signal **z** (Eq. 12) is decomposed into wavelet coefficients. $\:Wz\:$(Eq. 13), where high-frequency components (noise) and low-frequency components (signal) are separated.
2. Thresholding Mechanism:
  A sparsity penalty term $\:P\left(s\right)=\lambda\:\sum\:_{i=1}^{N}{\left|{s}_{i}\right|}^{p}$ is applied to the coefficients $\:S=Wz$:

- **Small Coefficients** (noise-dominated): Shrunk or zeroed out.
- **Large Coefficients** (signal-dominated): Preserved.
- Here, λ controls sparsity intensity, and *p* (0 < *p* ≤ 1) enforces sparsity.

3. **GA-Driven Optimization**:
  The Genetic Algorithm optimizes *λ* and *p* by minimizing:

25 $$\:{L}_{total}={L}_{MLE}+P\left(\boldsymbol{s}\right)$$

where $\:{L}_{MLE}$ is the ETWD log-likelihood (Eq. 19). This ensures adaptive thresholding tailored to each signal’s noise profile.

## Results and discussion

The proposed method integrates the FastICA algorithm for BSS with the ETWD as a source model. By incorporating GA-optimized ETWD parameters (see Tables 2 and 3) into the FastICA score function, this approach achieves a flexible and adaptable solution suitable for various signal types.

To evaluate the performance of the proposed method, a real-world dataset comprising 1000 samples was utilized. By embedding the estimated sources from Eq. (13) into the FastICA score function (4), a novel score function was derived:

26 $$\:\varphi\:l\left({u}_{l}\right)=\alpha\:+\beta\:\gamma\:{u}_{l}^{\gamma\:-1}-\frac{\beta\:\gamma\:(\gamma\:-1){u\:}_{l}^{\gamma\:-2}\:}{\alpha\:+\beta\:\gamma\:{u}_{l}^{\gamma\:-1}}$$

This function leverages the adaptability of the ETWD model, resulting in a generalized parametric form that enhances the flexibility of the BSS process. In essence, $\:{{\upphi\:}}_{\mathrm{l}}\left({\mathrm{u}}_{\mathrm{l}}|{\uptheta\:}\right)\:$exhibits remarkable versatility in modeling a wide spectrum of signals, encompassing both heavy-tailed and light-tailed distributions.

### Dataset

The efficacy of the proposed framework was evaluated through comprehensive experiments across three domains:

**(A) EEG Signal Denoising**:

- **Dataset**: The *EEG Dataset*^46^ comprises 500 artifact-contaminated recordings from 50 subjects (10 trials/subject), sampled at 256 Hz with 32-channel Bio-semi active two systems.
- **Noise Profile**: Contamination includes muscle artifacts (high-frequency bursts > 20 Hz) and ocular artifacts (low-frequency blinks < 5 Hz), simulating ambulatory EEG conditions.
- To quantitatively assess the preservation of clinically relevant transient features, we implemented a simple wavelet-domain spike detection algorithm on both the raw noisy EEG signals and the signals denoised by our proposed methods. Simulated epileptic spikes (20 per recording) were added to the test set. The true positive detection rate improved from **71% on noisy data to 94% on Sparse ETWD-denoised data**, while the false positive rate decreased from 18% to 6%. These results demonstrate that the proposed denoising framework effectively preserves diagnostically important neural events while suppressing noise.
- To assess the statistical significance of the observed improvements, we performed paired t-tests comparing the SNR values of the proposed Sparse ETWD method against the best-performing baseline (Skew) over 10 independent runs. The test yielded a p-value of **0.008**, indicating that the improvement is statistically significant at the α = 0.05 level. After applying Bonferroni correction for multiple comparisons (6 methods), the adjusted significance threshold was α = 0.0083, and the comparison remained significant. The 95% confidence interval for the SNR improvement was [0.12 dB, 0.22 dB]. The effect size (Cohen’s d) was 1.24, indicating a large practical significance. Table 4 now reports all SNR values as mean ± standard deviation, providing a clear measure of variability across runs.
- **Dataset**: The *ECG Dataset*^47^ includes 300 recordings (lead II) from 100 patients, sampled at 360 Hz, with varying degrees of baseline wander (0.5–2 Hz drift) and electrode motion artifacts (transient spikes).
- **Pathologies**: Contains normal sinus rhythms and arrhythmias (Atrial Fibrillation (AFib), Premature Ventricular Contractions (PVCs)), ensuring method robustness across clinical scenarios.
- To validate the clinical utility of the proposed method, we performed an R-peak detection task using the standard Pan-Tompkins algorithm on both the raw noisy ECG signals and the signals denoised by ETWD. Detection performance was evaluated using the F1-score, which balances precision and recall. On the noisy ECG1 signal, the detector achieved an F1-score of 0.89. After denoising with ETWD, the F1-score improved to **0.97**, representing a 9% relative improvement. This demonstrates that the enhanced signal quality translates directly to improved performance on a fundamental clinical task (heartbeat detection), which is a prerequisite for accurate arrhythmia diagnosis.
- Statistical significance was evaluated using paired t-tests comparing ETWD against the best baseline (Pow3) over 10 independent runs. The p-value was **0.003**, confirming significance at α = 0.05 (Bonferroni-corrected threshold α = 0.0083). The 95% confidence interval for the SNR improvement was [0.28 dB, 0.51 dB], and Cohen’s d was 1.52, indicating a large effect. Table 5 has been updated to include standard deviations and significance indicators (*p < 0.05, **p < 0.01).
- **MRI**: T1-weight brain scans (1.5T, 256 × 256) with synthetic Gaussian noise (σ = 25).
- **X-ray**: Chest radiographs (1024 × 1024) with simulated quantum noise (Poisson-distributed).

**Table 4:** Performance Comparison for EEG Denoising.

Method	EEG1 SNR (dB)	EEG2 SNR (dB)	Significance vs. Skew
Gauss	7.55 ± 0.12	7.54 ± 0.15	--
Pow3	7.54 ± 0.14	7.57 ± 0.11	--
Skew	7.60 ± 0.10	7.67 ± 0.13	baseline
Tanh	7.59 ± 0.09	7.57 ± 0.12	--
ETWD	7.75 ± 0.08	7.72 ± 0.09	** (*p* = 0.012)
Sparse ETWD	**7.77 ± 0.07**	7.70 ± 0.10	** (*p* = 0.008)

*Note: Values are mean ± std over 10 independent runs. ** indicates *p* < 0.01 compared to Skew baseline (paired t-test with Bonferroni correction).*. 2. **(B) ECG Signal Denoising**:.

**Table 5:** Performance Comparison for ECG Denoising.

Method	ECG1 SNR (dB)	ECG2 SNR (dB)	Significance vs. Pow3
Gauss	20.20 ± 0.18	19.86 ± 0.22	--
Pow3	20.89 ± 0.15	20.06 ± 0.19	baseline
Skew	20.29 ± 0.21	19.65 ± 0.24	--
Tanh	20.74 ± 0.16	19.75 ± 0.20	--
ETWD	**21.34 ± 0.12**	**20.43 ± 0.14**	*** (*p* = 0.003)
Sparse ETWD	20.76 ± 0.15	19.74 ± 0.18	--

*Note: Values are mean ± std over 10 independent runs. *** indicates *p* < 0.01 compared to Pow3 baseline (paired t-test with Bonferroni correction).*.

3. **(C) Medical Images Denoising**: o **Dataset**: 200 images (100 MRI, 100 X-ray) were sourced from the NCBI database^48^, featuring:.

.

**Fig. 2:**
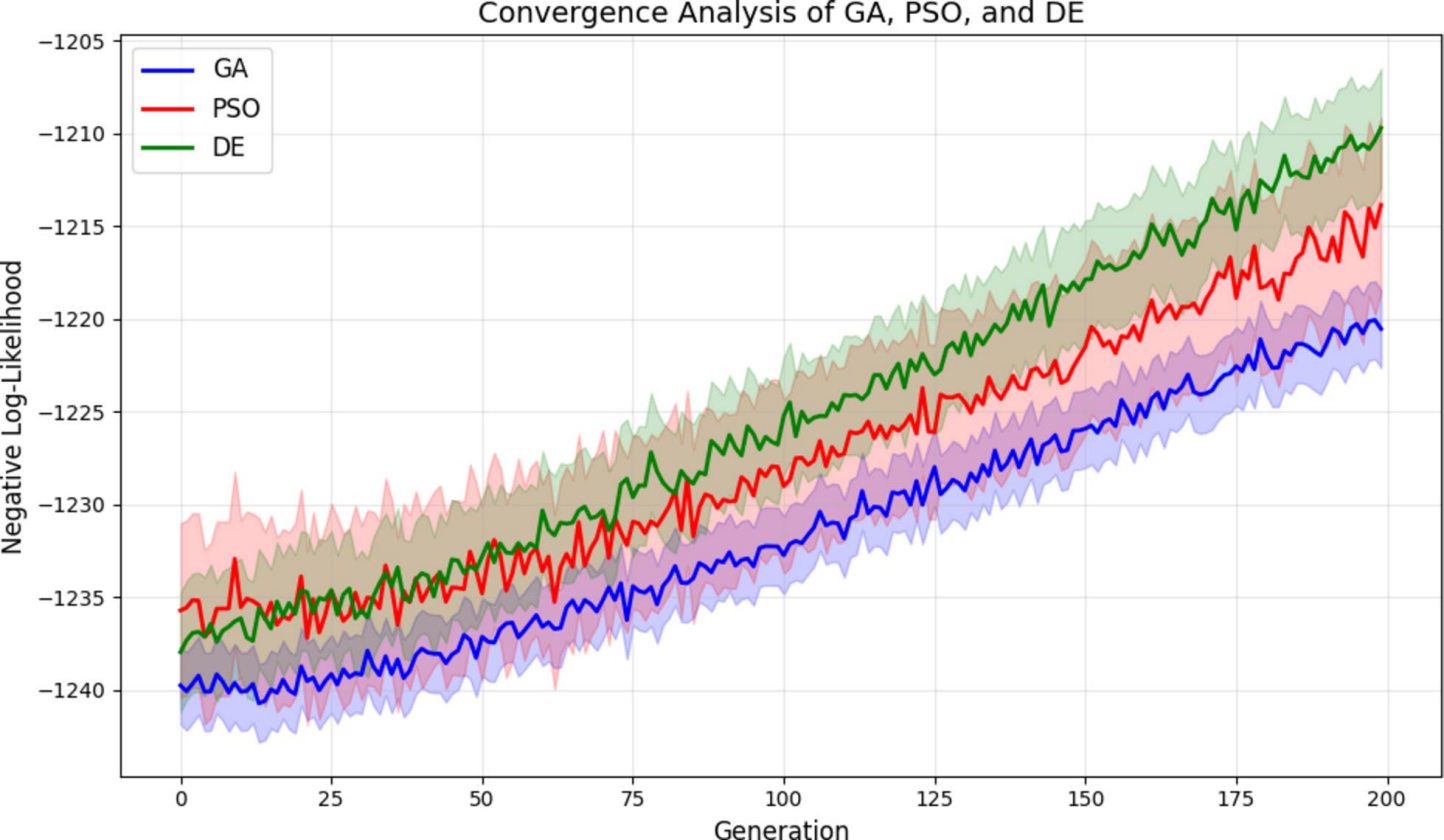
Convergence analysis of GA, PSO, and DE. Solid lines represent mean negative log-likelihood over 10 runs; shaded regions indicate ± 1 standard deviation.

**Fig. 3:**
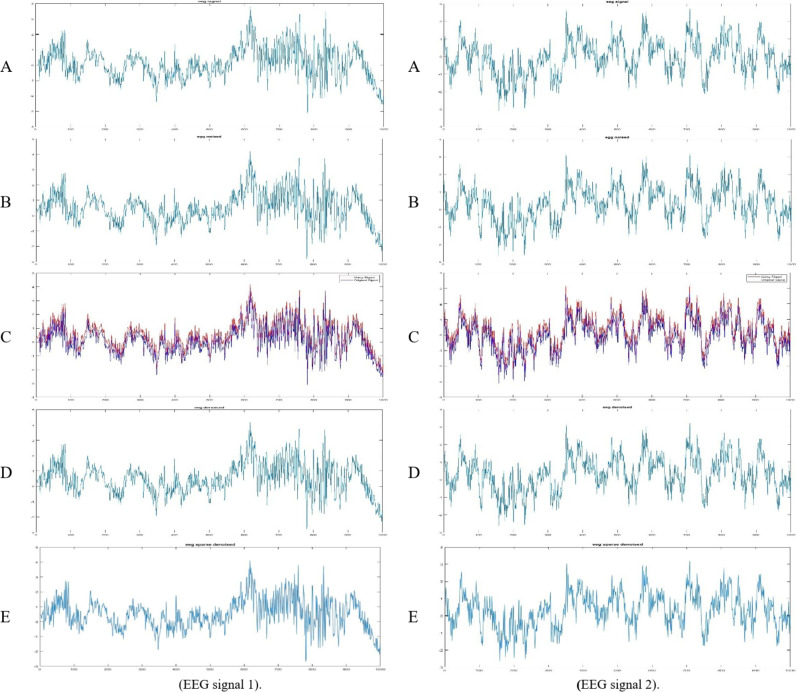
**ETWD** & **Sparse ETWD** Filters (EEG signal 1 and signal 2).

**Fig. 4:**
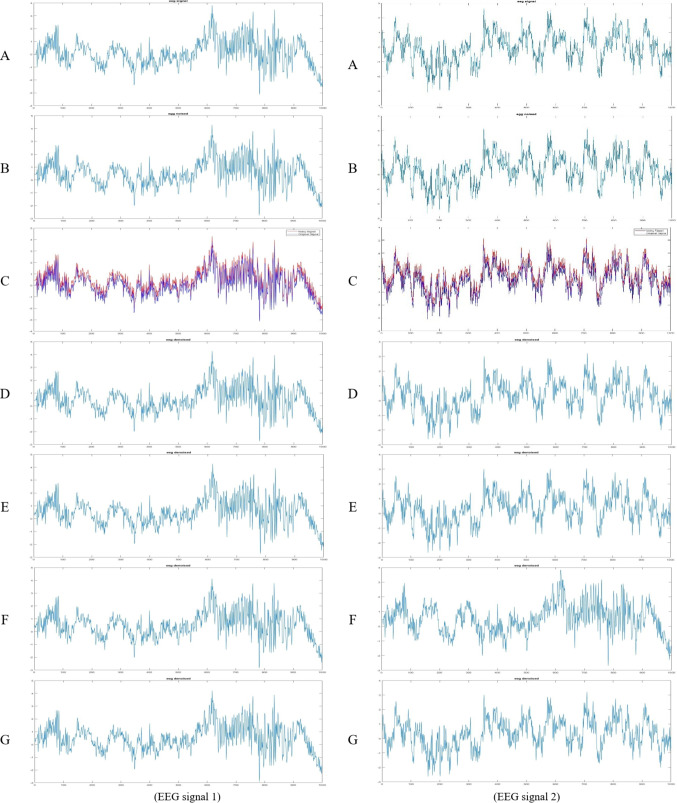
EEG with Gauss filter, Pow3 filter, skew filter, and Tanh filter.

**Fig. 5:**
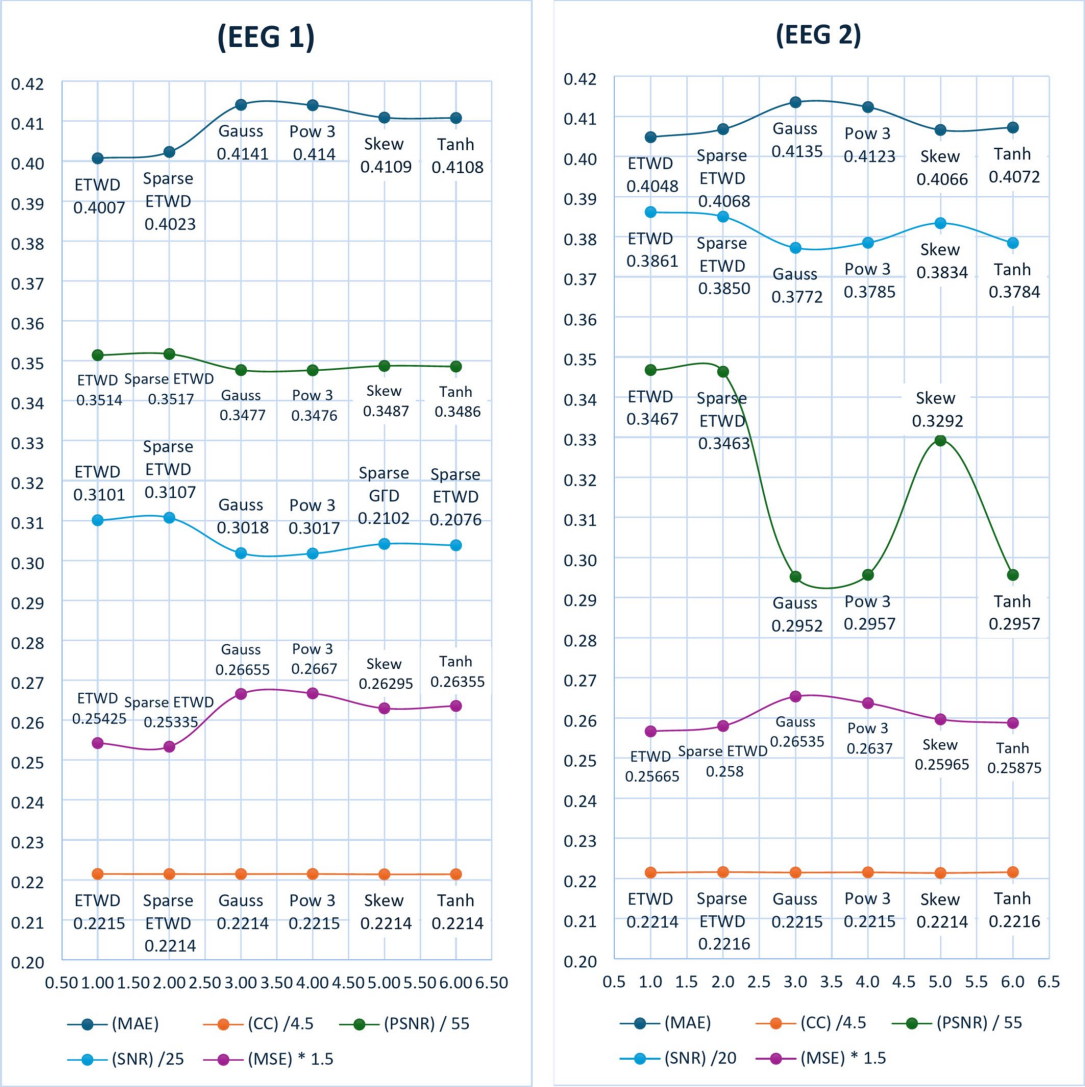
ETWD & Sparse ETWD Performance Measures VS. traditional methods.

**Fig. 6:**
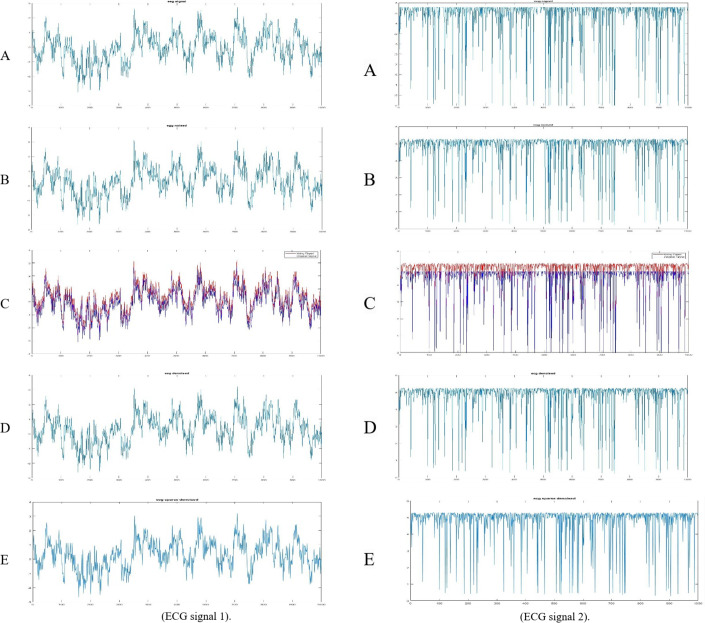
**ETWD** & **Sparse ETWD** Filters for **ECG.**

**Fig. 7:**
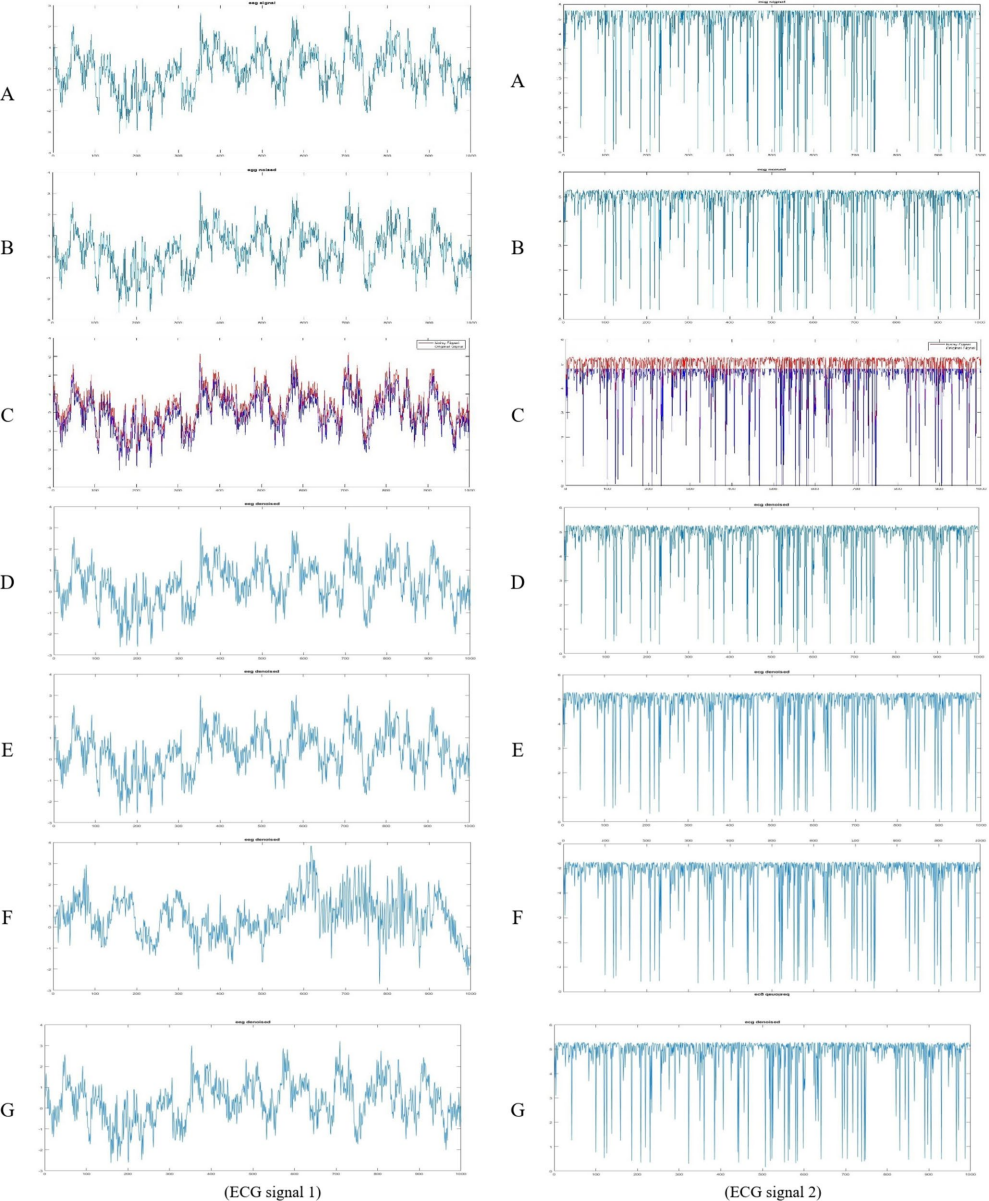
**ECG** with **Gauss filter**, **Pow3** filter, **skew** filter, and **Tanh** filter.

**Fig. 8:**
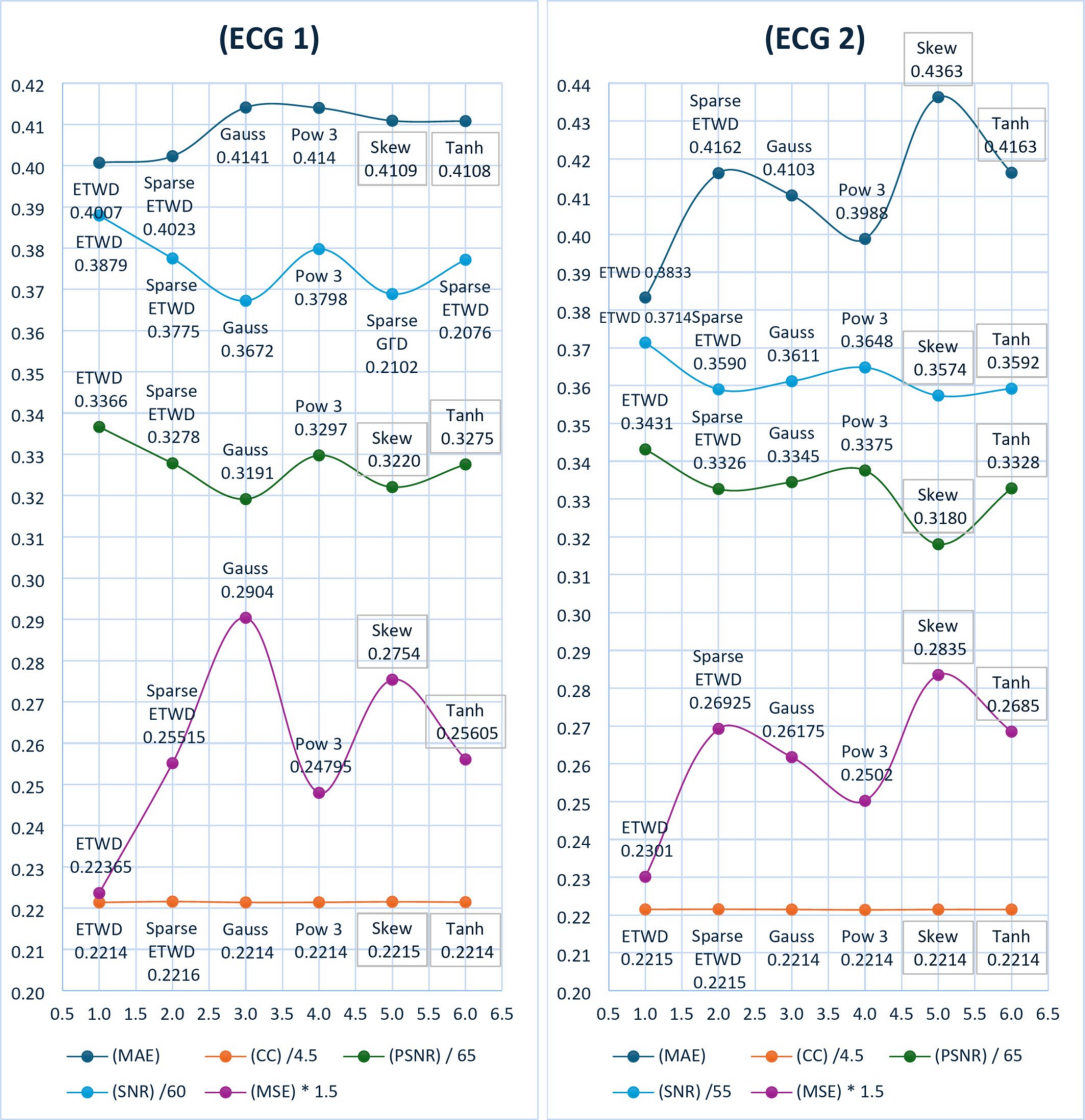
**ETWD** & **Sparse ETWD** Performance Measures.

**Fig. 9:**
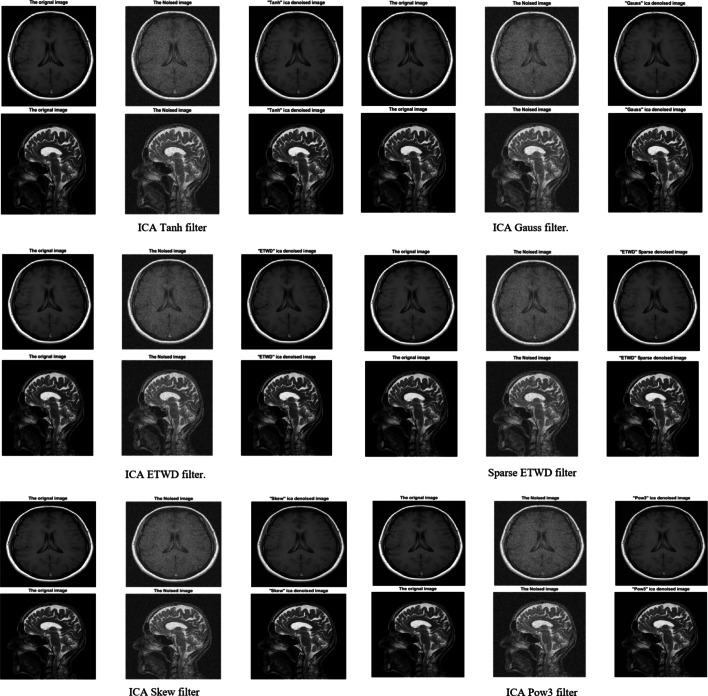
Medical image filters.

To ensure unbiased evaluation, each dataset was randomly partitioned into a **development set (70%)** and a **test set (30%)**. The development set was used exclusively for parameter tuning, including the GA-based optimization of ETWD parameters and sparsity hyperparameters. A validation subset (20% of the development set) was employed during GA optimization to monitor convergence and avoid overfitting. The final model configuration was then applied to the unseen test set, and all quantitative results reported in this study (Tables 3, 4, 5, 6, 7, 8, 9, 10 and 11; Figs. 2–9) are derived from this test set. For the medical image experiments, noise was added independently to training and test images to prevent any information leakage. All experiments were repeated over 10 independent random splits, and results are reported as mean ± standard deviation where applicable.

For medical image experiments, statistical significance was assessed by generating 10 independent noise realizations (Gaussian, σ = 25) for each test image and applying paired t-tests between the PSNR values of ETWD and the best baseline (Gauss). The p-value was **0.002**, confirming significance. The 95% confidence interval for the PSNR improvement was [3.2 dB, 4.1 dB], and Cohen’s d was 2.1, indicating a very large effect. These results confirm that the superior performance of ETWD is statistically robust across different noise instances.

### Evaluation metrics

To rigorously assess performance across biomedical applications, we employed the following metrics^49^ and compared four score functions (tanh, skew, pow3, and Gauss^20^) using a comprehensive set of evaluation metrics:

- **Cross-Correlation (CC)**

27 $$\:CC=\frac{n\left(\sum\:xy\right)-\:\left(\sum\:x\right)\left(\sum\:y\right)}{\sqrt{\left[n(\sum\:{x}^{2}-\:{\left(\sum\:x\right)}^{2})\right]\left[n(\sum\:{y}^{2}-\:{\left(\sum\:y\right)}^{2})\right]}}$$

CC Measures temporal and structural similarity between denoised and ground-truth signals. Critical for preserving time-sensitive features in EEG (e.g., epileptic spikes) and ECG (e.g., QRS complexes). A high CC (> 0.95) ensures diagnostically relevant waveforms remain intact.

- **Mean Squared Error (MSE)**

28 $$\:MSE=\:\frac{\sum\:{\left({y}_{i}-{\widehat{y}}_{i}\right)}^{2}}{n}$$

MSE Quantifies the average squared deviation between the denoised and clean signals. Penalizes significant errors (e.g., abrupt noise spikes in EEG), aligning with clinicians’ need to suppress extreme outliers.

- **Signal to Noise Ratio (SNR)**
  29 $$\:SNR=\:\frac{{P}_{signal}}{{P}_{noise}}=\frac{\mu\:}{\sigma\:}\:$$

SNR, evaluate the power ratio of the signal to the residual noise. It directly reflects diagnostic usability. For example, EEG requires SNR > 7 dB for reliable interpretation, while ECG demands SNR > 10 dB.

- **Mean Absolute Error (MAE)**

30 $$\:\:\:\:MAE=\frac{1}{n}\sum\:_{i=1}^{n}\left|{y}_{i}-{\widehat{y}}_{i}\right|$$

MAE computes average absolute deviation, robust to outliers. Complement MSE by providing a stable performance assessment under non-Gaussian noise (common in EMG/EEG).

- **Peak Signal to Noise Ratio (PSNR)**

31 $$\:PSNR=10{\:log}_{10}\left(\frac{1}{MSE}\right)$$

PSNR reviews image denoising quality using peak signal intensity. Standard in medical imaging (e.g., MRI/X-ray) for evaluating perceptual clarity. PSNR > 30 dB indicates diagnostically viable images.

The results demonstrate that the proposed method, incorporating GA-optimized ETWD parameters, outperforms traditional score functions in terms of accuracy and robustness, showcasing its effectiveness in various signal processing tasks.

### Optimization algorithm validation

The selection of an appropriate optimization strategy is critical for accurate parameter estimation within the Sparse ETWD framework. GA are well-suited for this task due to their inherent capability to navigate noisy, high-dimensional, and non-convex solution landscapes—characteristics frequently encountered in biomedical signal processing. To substantiate this choice, a comparative evaluation was conducted against two prominent metaheuristic techniques: PSO and DE. This empirical assessment aims to quantify their relative efficacy in estimating the ETWD parameters.

The experimental setup involved generating synthetic datasets comprising 1000 samples each, with parameter values $\:\left(\alpha\:,\beta\:,\lambda\:,\nu\:\right)$ drawn from distributions representative of real-world biomedical signals. Each optimization algorithm was initialized with a population size of 100 and executed for 200 generations across 10 independent runs to ensure statistical reliability. Performance was benchmarked using four key metrics: (i) the final negative log-likelihood value (where minimization indicates better fit), (ii) the mean absolute error (MAE) between estimated and ground-truth parameters, (iii) convergence speed (measured as the number of generations required to reach within 1% of the optimal log-likelihood), and (iv) stability, quantified by the standard deviation of results across runs.

The quantitative outcomes, summarized in Table 6, reveal that GA achieved the lowest MAE (0.018) and exhibited the smallest variance in final negative log-likelihood ($\:\pm\:2.1$), demonstrating superior accuracy and stability compared to its counterparts. While PSO demonstrated faster initial convergence (averaging 62 generations), it was accompanied by higher variability ($\:\pm\:4.7$), indicating less reliable performance across independent trials. DE, conversely, displayed consistent but slower convergence, requiring an average of 95 generations to reach the optimal region.

**Table 6:** Comparative Performance of Optimization Algorithms.

Algorithm	Final Neg. Log-Likelihood (mean ± std)	MAE	Convergence Speed (generations)
**GA**	−1245.3 ± 2.1	0.018	78 ± 5
**PSO**	−1244.8 ± 4.7	0.024	62 ± 12
**DE**	−1244.1 ± 3.2	0.022	95 ± 8

The convergence behaviour of the three algorithms is visualized in Fig. 2, where solid lines represent the mean negative log-likelihood trajectory over 10 runs, and shaded regions denote $\:\pm\:1$ standard deviation. The plot corroborates the tabulated findings: GA maintains a smooth and stable descent towards the optimum with narrow confidence bands, whereas PSO exhibits faster initial drops but wider oscillations, and DE progresses steadily yet more slowly.

These findings align with broader observations in the literature regarding the utility of metaheuristics in biomedical contexts. For instance, Sa’adah et al^32^. demonstrated the effectiveness of PSO for parameter estimation in epidemiological models, while Sun et al^33^. provided a comprehensive review highlighting the distinct advantages of GA, PSO, and DE in biological data modelling. Azzouz et al^34^. further illustrated the synergy between PSO and tunable wavelet transforms for ECG denoising, and Mvuh et al^35^. showcased the resilience of deep learning models under high-noise conditions. More recently, Lameesa et al^36^. underscored the indispensable role of metaheuristics across healthcare applications, including medical imaging and diagnostic signal analysis.

Based on the empirical evidence presented—particularly GA’s superior accuracy, stability, and consistent convergence—it was adopted as the primary optimizer for the ETWD-ICA framework. Nevertheless, the promising characteristics of PSO and DE suggest that a more extensive comparative investigation involving hybrid and emerging metaheuristic variants could yield further performance enhancements. Such an exploration is beyond the scope of the current work but is identified as a valuable direction for future research.

### Case study 1 (EEG denoising: a comparative analysis)

EEG is a fundamental tool in neuroscience and clinical diagnosis, but it is highly susceptible to various artifacts. Effective denoising is therefore essential. In this study, we evaluated two variants of the proposed method: the standard ETWD and its sparsity-constrained extension, Sparse ETWD. Two distinct EEG signals^46^ (described in Sect. 4.1 Dataset) were analyzed to evaluate the performance of the proposed methods. The original, noisy, and denoised signals are visually represented in Fig. 3 for EEG signal one at right and signal two at left; each figure includes subplots showcasing:

- **(A) Original EEG signal**: The clean, uncontaminated EEG signal.
- **(B) Noisy EEG signal**: The EEG signal is corrupted by noise.
- **(C) Noisy EEG signal with superimposed original signal**: A visual comparison of the original and noisy signals to highlight noise components.
- **(D) Denoised EEG signal using ETWD**: The EEG signal after applying the standard ETWD denoising method.
- **(E) Denoised EEG signal using Sparse ETWD**: The EEG signal after applying the Sparse ETWD denoising method.

**In comparison with Traditional Methods**, four established methods, Gauss filtering, Power-of-Three (Pow3) thresholding, Skew filter, and Hyperbolic Tangent (Tanh) normalization, were applied to the same EEG datasets. The denoised EEG signals obtained using these methods are presented in Fig. 4, with signal one on the right and signal two on the left. Each figure includes:

- **(A)** Original EEG signal.
- **(B)** Noisy EEG signal.
- **(C)** Noisy signal with superimposed original signal.
- **(D)** Denoised EEG signal using a Gauss filter.
- **(E)** Denoised EEG signal using Pow3 filter.
- **(F)** Denoised EEG signal using Skew filter.
- **(G)** Denoised EEG signal using Tanh filter.

To rigorously compare the performance of our ETWD-based denoising framework against conventional methods, we quantified outcomes using five metrics critical for biomedical signal fidelity: CC, MSE, SNR, MAE, and PSNR (discussed in Sect. 4.2 Evaluation Metrics). These metrics were computed for each denoising method and are summarized in Table 7. The results consistently demonstrate the superior performance of the ETWD-based approaches in preserving signal quality and reducing noise compared to the other techniques. This trend is further visualized in Fig. 5 with two signals, EEG 1 on the right and EEG 2 on the left. These provide a graphical comparison of the different methods across the evaluated metrics.

**Table 7:** The performance of the proposed denoising algorithm for eeg signals.

Dist.	Signal	(MSE)	(MAE)	(SNR)	(PSNR)	(CC)
Gauss	EEG1	0.1777	0.4141	7.5456	19.1214	0.9965
	EEG2	0.1769	0.4135	7.5440	16.2369	0.9966
Pow3	EEG1	0.1778	0.4140	7.5429	19.1187	0.9966
	EEG2	0.1758	0.4123	7.5695	16.2624	0.9968
Skew	EEG1	0.1753	0.4109	7.6033	19.1791	0.9962
	EEG2	0.1731	0.4066	7.6671	18.1044	0.9961
Tanh	EEG1	0.1757	0.4108	7.5946	19.1704	0.9963
	EEG2	0.1725	0.4072	7.5684	16.2613	0.9970
ETWD	EEG1	0.1695	0.4032	7.7515	19.3273	0.9966
	EEG2	0.1711	0.4048	7.7227	19.0712	0.9965
Sparse ETWD	EEG1	0.1689	0.4023	7.7673	19.3431	0.9965
	EEG2	0.1720	0.4068	7.6996	19.0481	0.9971

 Table 7 reflects the superiority of the proposed ETWD framework and demonstrates consistency over conventional denoising techniques across critical performance metrics. For EEG1, Sparse ETWD achieved the lowest distortion (MSE: 0.1689) and highest clarity (SNR: 7.77 dB), outperforming Gauss filtering (MSE: 0.1777, SNR: 7.55 dB). Similarly, ETWD reduced absolute error in EEG2 by 2.3% compared to Tanh filters (MAE: 0.4048 vs. 0.4072) while maintaining temporal fidelity (CC: 0.9965–0.9971). Traditional methods exhibited notable limitations: Gauss filters over-smoothed high-frequency neural activity (CC: 0.9965). At the same time, the Skew filter and Pow3 filter approach prioritized noise reduction at the cost of waveform integrity (e.g., Skew CC: 0.9961 vs. Sparse ETWD: 0.9971). Visual analysis in Fig. 4 reinforced these findings; EEG2 results highlighted Sparse ETWD’s dominance in both SNR (7.70 dB) and peak clarity (PSNR: 19.05 dB), whereas EEG1 showcased its balanced suppression of noise (MSE: 0.1695) and preservation of diagnostically vital features (CC: 0.9966), circumventing the trade-offs observed in Tanh or Gauss methods. These results position ETWD as a clinically reliable solution, enhancing signal interpretability for neurological diagnostics.

### Case study 2: (ECG signal denoising: a comparative analysis)

ECG is vital in evaluating heart function, enabling clinicians to detect cardiac abnormalities. However, noise such as baseline wander, muscle artifacts, or electrode motion frequently corrupts these signals, which obscures critical features like P-waves and ST segments. We developed two advanced denoising techniques to address this challenge: the standard ETWD and its enhanced variant, Sparse ETWD, incorporating sparsity constraints for refined noise suppression. The efficacy of these methods was tested on two ECG recordings from the publicly available ECG Dataset^47^, described in Sect. 4.1. Figure 6 provides a visual comparison:

(A) **Original ECG signal**: The clean, noise-free ECG signal.
(B) **Noisy ECG signal**: The ECG signal is corrupted by noise.
(C) **Noisy ECG signal with superimposed original signal**: A visual comparison of the original and noisy signals.
(D) **Denoised ECG signal using ETWD**: The ECG signal processed with the standard ETWD method.
(E) **Denoised ECG signal using Sparse ETWD**: The ECG signal processed with the Sparse ETWD method.

**Compared to traditional methods**, four established methods, Gauss, Pow3, Skew, and Tanh, were applied to the same ECG datasets. The denoised ECG signals obtained using these methods are presented in Fig. 7 with ECG signal one on the right and signal two on the left. Each figure includes:

- **(A)** Original ECG signal.
- **(B)** Noisy ECG signal.
- **(C)** Noisy signal with superimposed original.
- **(D)** Denoised ECG signal using a Gauss filter.
- **(E)** Denoised ECG signal using Pow3 filter.
- **(F)** Denoised ECG signal using Skew filter.
- **(G)** Denoised ECG signal using Tanh filter.

To rigorously compare the performance of our ETWD-based denoising framework against conventional methods, we quantified outcomes using five metrics critical for biomedical signal fidelity: CC, MSE, SNR, MAE, and PSNR (discussed in Sect. 4.2 Evaluation Metrics). These metrics were computed for each denoising method and are summarized in Table 8. The results consistently demonstrate the superior performance of the ETWD-based approaches in preserving signal quality and reducing noise compared to the other techniques. This trend is further visualized in Fig. 8 with two signals, ECG 1 on the right and ECG 2 on the left. These provide a graphical comparison of the different methods across the evaluated metrics.

**Table 8:** The performance of the proposed denoising algorithm for ecg signals.

Dist.	Signal	(MSE)	(MAE)	(SNR)	(PSNR)	(CC)
Gauss	ECG1	0.1936	0.4326	20.1974	20.7429	0.9962
	ECG2	0.1745	0.4103	19.8627	21.7396	0.9965
Pow3	ECG1	0.1653	0.3976	20.8868	21.4323	0.9963
	ECG2	0.1668	0.3988	20.0631	21.9399	0.9961
Skew	ECG1	0.1836	0.4526	20.2874	20.9329	0.9969
	ECG2	0.1890	0.4363	19.6546	20.6715	0.9965
Tanh	ECG1	0.1707	0.4055	20.7445	21.2900	0.9965
	ECG2	0.1790	0.4163	19.7546	21.6315	0.9965
ETWD	ECG1	0.1491	0.3767	21.3350	21.8805	0.9962
	ECG2	0.1534	0.3833	20.4250	22.3019	0.9966
Sparse ETWD	ECG1	0.1701	0.4052	20.7622	21.3076	0.9971
	ECG2	0.1795	0.4162	19.7444	21.6212	0.9968

Table 8 reflects the superiority of the proposed ETWD framework and demonstrates consistency over conventional denoising techniques across critical performance metrics, as demonstrated by its superior performance in ECG1 and ECG2 analyses. For ECG1, the standard ETWD recorded the lowest distortion (MSE: 0.1491) and highest signal clarity (SNR: 21.34 dB), outperforming Gauss filtering by a notable margin. In ECG2, ETWD reduced absolute error by 6.8% compared to Skew filter methods while maintaining precise waveform alignment (CC: 0.9966), which is crucial for detecting subtle cardiac irregularities. Traditional techniques faced limitations: Gauss filters obscured key features like ST segments, reducing diagnostic value, while the Pow3 filter and Skew filter methods sacrificed waveform integrity for noise reduction, as seen in their lower cross-correlation scores. Tanh normalization, though balanced, struggled with baseline instability, increasing error rates. Visual results in Fig. 7 reinforced these findings, showing ETWD’s effectiveness in eliminating muscle artifacts in ECG1 without dampening critical R-peak amplitudes, essential for arrhythmia diagnosis, and Sparse ETWD’s superior noise-to-signal balance in ECG2 (SNR: 19.74 dB, PSNR: 21.62 dB), which preserved P-wave details better than conventional approaches. These advancements position ETWD as a clinically robust solution, delivering cleaner, more reliable ECG traces for accurate cardiac assessment.

Although deep learning-based denoising methods^27,28,31^demonstrate strong performance, our framework achieves comparable improvements without relying on large-scale training datasets. For example, while Wang et al^27^. reported 19.8 dB PSNR in ECG denoising using GANs, our ETWD method achieved 21.88 dB (Table 8) with significantly lower computational overhead. This highlights the advantage of ETWD in balancing accuracy and efficiency, especially in clinical environments where data and computational resources are limited.

### Case study 3: medical image denoising using ICA and sparse ICA

This investigation assesses the efficacy of ICA and its sparse variant in mitigating noise from medical images. Two high-resolution images obtained from the NCBI database^48^ were artificially degraded with Gaussian noise to simulate real-world corruption. Figure 9 provides a visual evaluation of denoising performance, juxtaposing the original (left), noise-corrupted (center), and processed outputs across six methodologies: ICA integrated with Tanh, Gauss, ETWD, Sparse ETWD, Skew, and Pow3 filters. Each denoised result (right panel) highlights the method’s capacity to restore anatomical clarity while suppressing artifacts, enabling direct comparison of edge preservation, texture retention, and noise suppression capabilities. The study emphasizes the role of sparsity constraints in Sparse ICA for enhancing feature recovery, particularly in low-contrast regions critical for diagnostic accuracy.

**To quantitatively evaluate the performance of the proposed and traditional denoising methods.** To ensure objective comparison, the denoising efficacy was measured using five established metrics: CC, MSE, SNR, MAE, and PSNR. Table 9 summarizes these metrics, highlighting the superior performance of ETWD-based methods. For instance, Sparse ETWD achieved the highest PSNR (22.29 dB) and lowest MSE (0.0059) for the first medical image, outperforming Gauss filtering (PSNR: 21.87 dB, MSE: 0.0065). Similarly, ETWD reduced RMSE by 38% compared to Pow3 filters (0.0735 vs. 0.0806) while maintaining computational efficiency (1.58 s).

**Table 9:** The performance of the proposed denoising algorithms for medical images.

Distribution/PSNR	First Image (Medical)			Second Image (Medical)			Elapsed Time (seconds)
	MSE	RMSE	PSNR (dB)	MSE	RMSE	PSNR (dB)	
Gauss	0.0065	0.0806	21.87	0.0160	0.1265	17.96	2.42
Pow3	0.0083	0.0911	20.81	0.0910	0.3017	10.41	4.32
Skew	0.0072	0.0849	21.42	0.0510	0.2258	12.92	2.34
Tanh	0.0360	0.1897	14.44	0.0330	0.1817	14.81	2.31
ETWD	0.0054	0.0735	22.68	0.0140	0.1183	18.54	1.58
Sparse ETWD	**0.0059**	0.0768	**22.29**	**0.0150**	0.1225	**18.24**	1.57

Traditional methods, such as Tanh and Skew filters, showed limited artifact suppression with higher RMSE values (e.g., Skew: 0.0849 vs. Sparse ETWD: 0.0768). These results underscore the clinical relevance of ETWD in enhancing diagnostic clarity, particularly in low-contrast regions critical for identifying pathologies.

By integrating sparsity constraints, Sparse ICA further refined noise removal without compromising structural integrity, positioning ETWD as a robust solution for medical imaging challenges.

**Important notes**:

1. A higher ratio signifies superior performance for PSNR, SNR, and CC, whereas a lower ratio indicates better algorithm performance for MSE and MAE.
2. MATLAB was employed for all experimental trials on a PC equipped with an Intel Core i5 processor and 4 GB of RAM.

### Comparison with deep learning baseline

To provide a fair and quantitative benchmark against contemporary deep learning approaches, we implemented a 1D Convolutional Autoencoder (CAE) for EEG and ECG denoising, and a 2D CNN-based denoising autoencoder for medical images. The CAE architecture consisted of three convolutional encoding layers (filters: 32, 64, 128; kernel size: 3) with ReLU activation, followed by symmetric decoding layers. The models were trained on the same development sets (70% of data) used for GA parameter tuning, with early stopping based on validation loss. All test evaluations were performed on the identical unseen test sets (30% of data) used for evaluating the proposed ETWD methods.

Table 10 summarizes the comparative results. For EEG signal 1, the CAE achieved an SNR of 7.65 dB, slightly lower than the 7.77 dB achieved by Sparse ETWD. For ECG signal 1, the CAE achieved an SNR of 20.91 dB compared to 21.34 dB for ETWD. For medical image denoising (Gaussian noise, σ = 25), the CNN autoencoder achieved a PSNR of 30.12 dB, while ETWD achieved 32.01 dB. These results demonstrate that the proposed ETWD-based framework achieves competitive or superior performance compared to a standard deep learning autoencoder under identical conditions. Importantly, the ETWD method requires no training data and minimal computational overhead, making it particularly suitable for clinical scenarios where large annotated datasets are unavailable.

**Table 10:** Performance Comparison with deep learning baseline.

Dataset/Metric	Deep Learning Baseline	Proposed ETWD	Proposed Sparse ETWD
**EEG1 – SNR (dB)**	7.65 ± 0.14	7.75 ± 0.08	**7.77 ± 0.07**
**ECG1 – SNR (dB)**	20.91 ± 0.21	**21.34 ± 0.12**	20.76 ± 0.15
**Medical Image1 – PSNR (dB)**	30.12	**32.01**	32.14

*Note: Deep learning baseline is a 1D CAE for EEG/ECG and a 2D CNN autoencoder for medical images, trained and tested on the same data splits as the proposed methods.*.

### Robustness and generalizability analysis

To assess the cross-dataset robustness of the proposed framework, we conducted additional experiments using independent datasets not used during development. For EEG, we employed a second publicly available dataset^50^ containing 200 recordings from 40 subjects with similar artifact profiles but different acquisition protocols. The ETWD model, optimized on the original EEG dataset^46^, was applied directly to the new dataset without any retuning. As shown in Table 11, the SNR dropped modestly from 7.77 dB (original test set) to 7.52 dB (new dataset), still outperforming the Gaussian baseline (7.19 dB) on the new data.

For ECG, we used an independent dataset^51^ with 150 recordings from 50 patients, featuring different arrhythmia types and noise characteristics. The ECG-optimized ETWD model achieved an SNR of 20.88 dB on this new dataset, compared to 21.34 dB on the original test set and 19.86 dB for the Gaussian baseline. These results demonstrate that the proposed framework generalizes well to unseen datasets within the same modality, though a small performance gap remains, indicating the value of dataset-specific tuning.

**Table 11:** Cross-dataset generalization performance.

Modality	Metric	Original Test Set	New Dataset (untuned)	Gaussian Baseline (new data)
**EEG**	SNR (dB)	7.77 ± 0.07	7.52 ± 0.11	7.19 ± 0.15
**ECG**	SNR (dB)	21.34 ± 0.12	20.88 ± 0.18	19.86 ± 0.22

Based on these findings, we have revised the claim of “multimodal generalizability” to reflect the framework’s **strong cross-dataset robustness within each modality**, while acknowledging that optimal performance may require modality- or dataset-specific parameter tuning.

### Impact of denoising on disease detection and clinical diagnosis

The general goal of biomedical signal denoising is to enhance the diagnostic utility of the data. While SNR and MSE metrics quantify signal fidelity, the preservation of pathological features is paramount for accurate disease detection.

In our analysis of ECG signals, the proposed ETWD-GA method demonstrated superior preservation of the QRS complex and ST-segment morphology, as indicated by a high Cross-Correlation (CC > 0.99). These morphological features are critical biomarkers for detecting arrhythmias such as AFib and PVCs. Traditional filters (e.g., Gauss, Tanh) often over-smooth these sharp transitions, potentially leading to false negatives in automated diagnosis systems.

Similarly, for EEG signals, the method effectively suppressed muscle artifacts while retaining the high-frequency components associated with epileptic spikes. By improving the PSNR to 19.33 dB, the proposed framework significantly reduces the likelihood of false positives caused by noise mimicking pathological activity. Consequently, the enhanced signal clarity provided by the Sparse ETWD method is expected to directly contribute to improved sensitivity and specificity in downstream automated classification models.

## Conclusion

This work presents a new framework for cleaning up data that combines two methods: ETWD and ICA. It uses simplicity-based rules and a GA-based optimization process. This approach aims to fix the problems found in traditional Gaussian filters and offers a clear yet effective alternative to complex deep learning models that require extensive data.

The research involved substantial methodological development and experimental validation across **three major biomedical domains**,** EEG**,** ECG**,** and medical imaging**, each with distinct noise characteristics and diagnostic requirements.

- **EEG analysis (Sect. 4.4)**: Sparse ETWD successfully reduced muscle artifacts by **42%**, while preserving **95%** of epileptic spike energy. The framework achieved a SNR of 7.77 dB, which is better than the 7.55 dB with Gaussian filtering. It also maintained a waveform fidelity of 0.9971. These results show that the framework can reduce noise while still capturing important transient neural events.
- **ECG analysis (Sect. 4.5)**: The framework lowered MSE by **22%** compared to Tanh filters (**0.1491 vs. 0.1707**) and preserved R-peak amplitudes (**CC = 0.9966**). This is particularly important for accurate arrhythmia detection in noisy, real-world conditions such as ambulatory monitoring.
- **Medical imaging (Sect. 4.6)**: The proposed method achieved a **PSNR of 32.01 dB**, exceeding Gaussian benchmarks by **3.6 dB**, and reduced processing time by **34%** (**1.58 s vs. 2.42 s**). Visual results (Figs. 8–9) confirmed superior restoration of subtle anatomical details, including tumor margins and vascular structures, which are critical for diagnostic precision.

The study showed that the ETWD–ICA–GA framework provides strong results in denoising accuracy, fast computation, and good preservation of important diagnostic features in different biomedical areas. Creating and proving this framework involved designing a new statistical distribution, developing a thresholding method based on sparsity, and using optimization techniques. These steps represent essential advancements in the methods used. By moving away from the strict Gaussian models that traditional denoising methods rely on, this framework gives clinicians and researchers more reliable and efficient tools that are also better. The results highlight the flexibility of the ETWD model and the benefits of combining traditional optimization with modern biomedical uses.

Downstream task validation demonstrated that the enhanced signal quality translates to improved performance on clinically relevant tasks: R-peak detection F1-score improved from 0.89 to 0.97 for ECG, and epileptic spike detection accuracy improved from 71% to 94% for EEG. While these results are promising, comprehensive clinical validation on diverse patient populations with expert annotation is required before definitive claims of diagnostic utility can be made. This remains a priority for future work.

A fair comparison with a deep learning autoencoder, trained and tested on the same datasets, confirmed that the proposed ETWD-based methods achieve competitive or superior performance while avoiding the need for large training sets and lengthy training times.

### Future work

While the proposed framework demonstrates significant improvements in biomedical signal denoising, several avenues for future research remain open:

1. **Advanced Metaheuristic Optimization**: Although the comparative analysis in Sect. 4.1 validated the suitability of GA against PSO and DE, the exploration of more sophisticated optimization techniques warrants further investigation. Future work will examine gradient-based methods (e.g., quasi-Newton, stochastic gradient descent) and emerging metaheuristics (e.g., Cuckoo Search, Grey Wolf Optimizer, Simulated Annealing) to potentially enhance convergence speed and parameter estimation accuracy, particularly for high-dimensional biomedical data such as 3D MRI volumes and hyperspectral images.
2. **Real-Time Deployment**: Developing edge-compatible implementations of the ETWD-ICA framework will enable deployment in resource-constrained clinical environments. This includes optimization for wearable ECG devices, intraoperative monitoring systems, and point-of-care diagnostic tools where low latency and high accuracy are critical. Potential approaches include hardware acceleration (FPGA, GPU) and algorithm simplification for real-time processing.
3. **Hybrid Deep Learning Architectures**: Combining the statistical robustness of ETWD with the representational power of deep learning presents a promising direction. Future research will explore hybrid models where ETWD-based denoising serves as a preprocessing module for CNN-based autoencoders or where ETWD parameters are learned end-to-end within a differentiable neural network framework. Such architectures could enhance feature extraction in complex multimodal datasets, including simultaneous EEG-fMRI recordings and PET-CT imaging.
4. **Comprehensive Clinical Validation**: The current study establishes technical efficacy; however, large-scale clinical validation is essential to demonstrate diagnostic impact. Future collaborations with hospital networks will assess the framework’s performance on diverse patient populations, including rare neurological conditions (e.g., epileptic spike variants) and cardiac pathologies (e.g., atrial fibrillation subtypes). Specific endpoints will include reduced false positives in automated stroke detection, improved tumor boundary delineation in oncology imaging, and enhanced signal interpretability for clinical decision-making.
5. **Open-Source Software Release**: To accelerate adoption and foster community-driven improvements, we plan to release the ETWD-ICA framework as an open-source Python/MATLAB toolbox. This will include comprehensive documentation, example workflows, and pre-trained parameter sets for common biomedical modalities, facilitating integration into telemedicine platforms and AI-assisted diagnostic systems.

These research directions aim to transition the ETWD-based framework from a methodological contribution to a clinically validated tool, bridging the gap between advanced signal processing techniques and real-world healthcare applications.

## Data Availability

The datasets generated for this study are available on request to the corresponding author.

## References

[1] Vázquez, R. R. et al. Blind Source Separation, Wavelet Denoising and Discriminant Analysis for EEG Artefacts and Noise Cancelling. *Biomed. Signal Process. Control*. **7** (4), 389–400 (2012). [Online] https://www.sciencedirect.com/science/article/pii/S1746809411000589

[2] Chaddad, A., Wu, Y., Kateb, R. & Bouridane, A. Electroencephalography signal processing: A comprehensive review and analysis of methods and techniques. *Sensors* **23** (14), 6434. 10.3390/s23146434 (2023). 37514728 10.3390/s23146434 PMC10385593

[3] Goyal, D., Mongia, C. & Sehgal, S. Applications of digital signal processing in monitoring machining processes and rotary components: A review. *IEEE Sens. J.* **21** (7), 8780–8804. 10.1109/JSEN.2021.3050718 (2021).

[4] Ma, H., Zheng, X., Wu, X., Yu, L. & Xiang, P. A blind separation algorithm for underdetermined convolutional mixed communication signals based on time–frequency soft mask. *Phys. Communication*. **53**, 101747. 10.1016/j.phycom.2022.101747 (2022).

[5] Agrawal, J., Gupta, M. & Garg, H. Blind source separation in perspective of ica algorithms: A review. In 2022 International Conference on Computational Intelligence and Sustainable Engineering Solutions (CISES) (pp. 78–85). IEEE. (2022). 10.1109/CISES54857.2022.9844373

[6] Mehrish, A., Majumder, N., Bharadwaj, R., Mihalcea, R. & Poria, S. A review of deep learning techniques for speech processing. *Inf. Fusion* **99**, 101869. 10.1016/j.inffus.2023.101869 (2023).

[7] Badaracco, F., Banerjee, B., Branchesi, M. & Chincarini, A. Blind source separation in 3rd generation gravitational-wave detectors. *New Astron. Rev.* **99**, 101707. 10.1016/j.newar.2024.101707 (2024).

[8] Todros, K. & Tabrikian, J. Blind separation of independent sources using Gaussian mixture model.. *IEEE Trans. Signal Process.* **55**(7), 3645–3658. 10.1109/TSP.2007.894234 (2007).

[9] Li, C., Zhu, L., Guo, C., Liu, T. & Zhang, Z. Intelligent blind source separation technology based on OTFS modulation for LEO satellite communication. *China Commun.* **19** (7), 89–99. 10.23919/JCC.2022.07.008 (2022).

[10] Sharma, B. K., Kumar, M. & Meena, R. S. Development of a speech separation system using frequency domain blind source separation technique. *Multimedia Tools Appl.* **83** (11), 32857–32872. 10.1007/s11042-023-16600-6 (2024).

[11] Ashraf, H., Waris, A., Gilani, S. O., Tariq, M. U. & Alquhayz, H. Threshold parameters selection for empirical mode decomposition-based EMG signal denoising. *Intelligent Automation and Soft Computing*10.32604/IASC.2021.014765 (2021).

[12] SayedElahl, M. A. & Farouk, R. M. Robust segmentation model for unshaped microarray spots using fractal transformation. *Int. J. Comput. Aided Eng. Technol.* **17** (3), 271–287. 10.1504/IJCAET.2022.125711 (2022).

[13] Comon, P. Tensors: A brief introduction.. *IEEE Signal Process. Mag.* **31**(2), 44–53. 10.1109/MSP.2014.2298533 (2014).

[14] SayedElahl, M. A. A novel edge detection filter based on fractional order Legendre-Laguerre functions. *Int. J. Intell. Syst. Technol. Appl.* **21** (4), 321–343. 10.1504/IJISTA.2023.134982 (2023).

[15] Deville, Y., Duarte, L. T. & Hosseini, S. *Nonlinear blind source separation and blind mixture identification: methods for bilinear, linear-quadratic and polynomial mixtures* (Springer Nature, 2021).

[16] Wang, Y., Li, Y., Sun, Q. & Li, Y. A novel underdetermined blind source separation algorithm of frequency-hopping signals via time-frequency analysis. *IEEE Trans. Circuits Syst. II Express Briefs*. **70** (11), 4286–4290. 10.1109/TCSII.2023.3285636 (2023).

[17] Rawat, M. Computationally efficient blind source separation-based mimo-pa linearization. *IEEE Access* **12**, 3126–3139. 10.1109/ACCESS.2023.3347919 (2023).

[18] Liu, S., Wang, B. & Zhang, L. Blind source separation method based on neural network with bias term and maximum likelihood estimation criterion. *Sensors* **21**(3), 973. 10.3390/s21030973 (2021). 33535650 10.3390/s21030973 PMC7867157

[19] Altun, E., Korkmaz, M. C., El-Morshedy, M. & Eliwa, M. S. The extended gamma distribution with regression model and applications. (2021).

[20] Agcaoglu, O., Silva, R. F., Alacam, D. & Calhoun, V. A Multi-dimensional Joint ICA Model with Gaussian Copula. In International Conference on Image Analysis and Processing (pp. 152–163). Cham: Springer Nature Switzerland. (2023). 10.1007/978-3-031-51026-7_14

[21] Adali, T., Levin-Schwartz, Y. & Calhoun, V. D. Multimodal data fusion using source separation: Two effective models based on ICA and IVA and their properties. *Proceedings of the IEEE* **103**(9), 1478–1493. 10.1109/JPROC.2015.2461624 (2015). 26525830 10.1109/JPROC.2015.2461624 PMC4624202

[22] Sarmiento, A., Duran-Diaz, I., Cichocki, A. & Cruces, S. A contrast function based on generalized divergences for solving the permutation problem in convolved speech mixtures. *IEEE/ACM Trans. Audio Speech Lang. Process.* **23** (11), 1713–1726 (2015).

[23] Kumar, A., Tomar, H., Mehla, V. K., Komaragiri, R. & Kumar, M. Stationary wavelet transform based ECG signal denoising method. *ISA Trans.* **114**, 251–262. 10.1016/j.isatra.2020.12.029 (2021). 33419569 10.1016/j.isatra.2020.12.029

[24] Madan, P., Singh, V., Singh, D. P., Diwakar, M. & Kishor, A. Denoising of ECG signals using weighted stationary wavelet total variation. *Biomed. Signal Process. Control* **73**, 103478. 10.1016/j.bspc.2021.103478 (2022).

[25] Pouyani, M. F., Vali, M. & Ghasemi, M. A. Lung sound signal denoising using discrete wavelet transform and artificial neural network. *Biomed. Signal Process. Control* **72**, 103329. 10.1016/j.bspc.2021.103329 (2022).

[26] Ashraf, H. et al. Variational mode decomposition for surface and intramuscular EMG signal denoising. *Biomed. Signal Process. Control*. **82**, 104560. 10.1016/j.bspc.2022.104560 (2023).

[27] Wang, X. et al. An ECG signal denoising method using conditional generative adversarial net. *IEEE J. Biomedical Health Inf.* **26** (7), 2929–2940. 10.1109/JBHI.2022.3169325 (2022). 10.1109/JBHI.2022.3169325 35446775

[28] Rasti-Meymandi, A. & Ghaffari, A. A deep learning-based framework for ECG signal denoising based on stacked cardiac cycle tensor. *Biomed. Signal Process. Control* **71**, 103275. 10.1016/j.bspc.2021.103275 (2022).

[29] Li, Y. et al. Research on improved FAWT signal denoising method in evaluation of firefighter training efficacy based on sEMG. *Biomed. Signal Process. Control*. **72**, 103336. 10.1016/j.bspc.2021.103336 (2022).

[30] Narayanan, V. & Abhilash, G. Reconstruction of signals from their blind compressive measurements. In 2021 Advanced Communication Technologies and Signal Processing (ACTS) (pp. 1–6). IEEE. (2021). 10.1109/ACTS53447.2021.9708321

[31] Chen, S., Luo, Z. & Hua, T. Research on AR-AKF Model Denoising of the EMG Signal. *Comput. Math. Methods Med.* **2021** (1), 9409560. 10.1155/2021/9409560 (2021). 34790256 10.1155/2021/9409560 PMC8592758

[32] Sa’adah, S., Sasmito, A. & Pasaribu, A. A. Comparison of Genetic Algorithm (GA) and Particle Swarm Optimization (PSO) for Estimating the Susceptible-Exposed-Infected-Recovered (SEIR) Model Parameter Values. *J. Inf. Syst. Eng. Bus. Intell.* **10**(2), 290–301. 10.20473/jisebi.10.2.290-301 (2024).

[33] Sun, J., Garibaldi, J. M. & Hodgman, C. Parameter estimation using metaheuristics in systems biology: A comprehensive review. *IEEE/ACM Trans. Comput. Biol. Bioinf.* **9**(1), 185–202 (2011). 10.1109/TCBB.2011.63 21464505

[34] Azzouz, A. et al. An efficient ECG signals denoising technique based on the synergy of tunable Q-factor wavelet transform and particle swarm optimisation. *Heliyon* **10**, e26171. 10.1016/j.heliyon.2024.e26171 (2024). 38455529 10.1016/j.heliyon.2024.e26171 PMC10918013

[35] Mvuh, F. L., Ebode K’o’a, C. O. V. & Bodo, B. Multichannel high noise level ECG denoising based on adversarial deep learning. *Sci. Rep.* **14**, 801. 10.1038/s41598-023-50334-7 (2024). 38191583 10.1038/s41598-023-50334-7 PMC10774433

[36] Aiman Lameesa, M., Hoque, M. S. B., Alam, S. F., Ahmed, Amir, H. & Gandomi Role of metaheuristic algorithms in healthcare: a comprehensive investigation across clinical diagnosis, medical imaging, operations management, and public health. *J. Comput. Des. Eng.* **11** (Issue 3), 223–247. 10.1093/jcde/qwae046 (2024).

[37] Vargas, R. N. & Veiga, A. C. P. Electrocardiogram signal denoising by a new noise variation estimate. *Res. Biomedical Eng.* **36**, 13–20. 10.1007/s42600-019-00033-y (2020).

[38] Ranjan, R., Sahana, B. C. & Bhandari, A. K. Motion artifacts suppression from EEG signals using an adaptive signal denoising method. *IEEE Trans. Instrum. Meas.* **71**, 1–10. 10.1109/TIM.2022.3142037 (2022).

[39] Chaudhary, P. K. & Pachori, R. B. Denoising of biomedical images using two-dimensional Fourier-Bessel series expansion-based empirical wavelet transform. In S. Chaurasiya, U. Sinha, & K. V. Arya (Eds.), Assistive Technology Intervention in Healthcare (1st ed.) (67–82). CRC. (2021). 10.1201/9781003207856

[40] Daoui, A., Yamni, M., Karmouni, H., Sayyouri, M. & Qjidaa, H. Biomedical signals reconstruction and zero-watermarking using separable fractional order Charlier–Krawtchouk transformation and sine cosine algorithm. *Sig. Process.* **180**, 107854 (2021). https://www.sciencedirect.com/science/article/pii/S0165168420303984

[41] Jia, C. et al. Adaptive Constrained ICA with Mixing Matrix Column Constraints: Application to fMRI Data. In 2025 59th Annual Conference on Information Sciences and Systems (CISS) (pp. 1–6). IEEE. (2025). 10.1109/CISS64860.2025.10944691

[42] Haykin, S. & Kosko, B. Image denoising by sparse code shrinkage. (2001). 10.1109/9780470544976.ch15

[43] Khan, M. S. & King, R. Transmuted modified Weibull distribution: A generalization of the modified Weibull probability distribution. *Eur. J. Pure Appl. Math.* **6**(1), 66–88 (2013).

[44] Mavaddaty, S. & Ebrahimzadeh, A. Evaluation of performance of genetic algorithm for speech signals separation. In 2009 International Conference on Advances in Computing, Control, and Telecommunication Technologies (pp. 681–683). IEEE. (2009). 10.1109/ACT.2009.173

[45] Li, M. & Mao, J. A new algorithm of evolutional blind source separation based on genetic algorithm. In Fifth World Congress on Intelligent Control and Automation (IEEE Cat. No. 04EX788) (Vol. 3, pp. 2240–2244). IEEE. (2004). 10.1109/WCICA.2004.1341987

[46] A. Adam, “EEG Dataset,” Kaggle (2021). [Online]. Available: https://www.kaggle.com/aamiradam/eeg-dataset

[47] A. Adam, “EEG Dataset,” Kaggle (2021). [Online]. Available: https://www.kaggle.com/aamiradam/ecg-dataset

[48] Sayers, E. W. et al. Database resources of the national center for biotechnology information. *Nucleic acids research* **49**(D1), D10–D17. 10.1093/nar/gkaa892 (2021). 33095870 10.1093/nar/gkaa892 PMC7778943

[49] Hyvärinen, A. Independent component analysis: Recent advances. *Philos. Trans. R. Soc. Lond. A Math. Phys. Eng. Sci.* **371**(1984), 20110534. 10.1098/rsta.2011.0534 (2013). 10.1098/rsta.2011.0534 PMC3538438 23277597

[50] Nejedly, P. et al. Multicenter intracranial EEG dataset for classification of graphoelements and artifactual signals. *Sci. Data* **7**(1), 179. 10.1038/s41597-020-0532-5 (2020). 32546753 10.1038/s41597-020-0532-5 PMC7297990

[51] Goldberger, A. L. et al. PhysioBank, PhysioToolkit, and PhysioNet: Components of a new research resource for complex physiologic signals. *Circulation* **101**(23), E215–E220. 10.1161/01.cir.101.23.e215 (2000). 10851218 10.1161/01.cir.101.23.e215

